# Multistate Gene Cluster Switches Determine the Adaptive Mitochondrial and Metabolic Landscape of Breast Cancer

**DOI:** 10.1158/0008-5472.CAN-23-3172

**Published:** 2024-06-26

**Authors:** Michela Menegollo, Robert B. Bentham, Tiago Henriques, Seow Q. Ng, Ziyu Ren, Clarinde Esculier, Sia Agarwal, Emily T.Y. Tong, Clement Lo, Sanjana Ilangovan, Zorka Szabadkai, Matteo Suman, Neill Patani, Avinash Ghanate, Kevin Bryson, Robert C. Stein, Mariia Yuneva, Gyorgy Szabadkai

**Affiliations:** 1 Department of Biomedical Sciences, University of Padova, Padova, Italy.; 2 Department of Cell and Developmental Biology, Consortium for Mitochondrial Research, University College London, London, United Kingdom.; 3 Department of Computer Sciences, University College London, London, United Kingdom.; 4 Department of Oncology, University College London Hospitals, London, United Kingdom.; 5 UCL Cancer Institute, University College London, London, United Kingdom.; 6 The Francis Crick Institute, London, United Kingdom.

## Abstract

Adaptive metabolic switches are proposed to underlie conversions between cellular states during normal development as well as in cancer evolution. Metabolic adaptations represent important therapeutic targets in tumors, highlighting the need to characterize the full spectrum, characteristics, and regulation of the metabolic switches. To investigate the hypothesis that metabolic switches associated with specific metabolic states can be recognized by locating large alternating gene expression patterns, we developed a method to identify interspersed gene sets by massive correlated biclustering and to predict their metabolic wiring. Testing the method on breast cancer transcriptome datasets revealed a series of gene sets with switch-like behavior that could be used to predict mitochondrial content, metabolic activity, and central carbon flux in tumors. The predictions were experimentally validated by bioenergetic profiling and metabolic flux analysis of ^13^C-labeled substrates. The metabolic switch positions also distinguished between cellular states, correlating with tumor pathology, prognosis, and chemosensitivity. The method is applicable to any large and heterogeneous transcriptome dataset to discover metabolic and associated pathophysiological states.

**Significance:**

A method for identifying the transcriptomic signatures of metabolic switches underlying divergent routes of cellular transformation stratifies breast cancer into metabolic subtypes, predicting their biology, architecture, and clinical outcome.

## Introduction

Evaluating patterns of metabolic adaptation during cancer development is essential to target tumor-specific vulnerabilities or therapy resistance (1, 2). Adaptation patterns can accompany transient alterations of cellular phenotypes or transitions in cellular states (3, 4). There has been recent interest in understanding metabolic adaptations during cell state transitions, revealing metabolic alterations at the metabolite and flux levels (1, 5–7). Metabolic measurements themselves however are insufficient to understand functional adaptation to the cellular state, unless linked to alterations in large-scale gene expression patterns (GEP), which almost invariably accompany cellular state transitions and bear important prognostic value (8–10). Functional metabolic adaptation thus could be more precisely evaluated by exploring the metabolic transcriptome—including expression of metabolic pathway components and nuclear encoded mitochondrial genes that together represent >15% of the genome—in association with cell state–specific large-scale GEPs.

Large patterns of alterations in cancer transcriptomes, often involving thousands of genes, arise both from genetic and environmental influences, determining the cellular phenotype and the associated cellular metabolic network (11). Earlier studies on cancer-related metabolic rearrangements mostly focused on the effect of cell-autonomous oncogenic signals on single or restricted groups of metabolic pathways (12, 13), delineating metabolic adaptations required for tumor growth, or hypoxia (14). However, recent whole genome transcriptome studies indicated that any tumor state, subtype, or phenotype can be identified by their specific and persistent transcriptional constellation (15–17). We argued that the unique transcriptional identity of tumor cells will inherently affect the whole metabolic transcriptome (8, 9, 18), and thus large-scale metabolic gene expression patterns will reflect *bona fide* adaptive transcriptional regulation of the metabolic phenotype associated with a specific tumor phenotype. To test this hypothesis, here we aimed to identify differential regulation patterns in the full metabolic transcriptome and link these quantitatively with the large-scale GEPs associated with different cancer cell states.

Stable transcriptional states have been shown to be modular, involving both activation and repression of gene sets (19), thus divergence between different states can be described as on and off switches of large gene sets in a bistate or multistate manner (20). As the gene sets modules are coregulated, they can be identified in an unbiased manner by searching for biclusters consisting of the highest positive (on switch) or negative (off switch) correlating clusters of transcripts in a subset of tumor samples. We have recently developed a massive correlated biclustering method (MCbiclust) to perform this task (21). Here we describe a pipeline based on this method for the discovery and further analysis of large gene expression switches, which can be used as a resource to analyze any large heterogeneous dataset. To demonstrate the workflow and the value of the resource, we use it for the discovery of metabolic and mitochondrial states (subtypes) of breast cancer. We show that the identified biclusters represent metabolic and bioenergetic switches, and the resulting gene sets can be used for important predictions about tumor architecture, biology, and clinically important features such as overall survival and chemosensitivity.

## Materials and Methods

### Cell lines

Human breast cancer cell lines MCF7, T47D, Hs578t, MDA436, and HCC1143 were purchased from ATCC. Their identity was confirmed through cell line authentication carried out by BMR Genomics by amplification of 23 STR loci (PowerPlex Fusion System kit, Promega). Cells were cultured in DMEM (#41966029), supplemented with 10% fetal bovine serum (FBS; Thermo Fisher Scientific) and Normocin (0.1 mg/mL, InvivoGen), and maintained in a 37°C incubator set with 5% CO_2_ and 95% humidity. Cell numbers for each experimental setting were counted using a hemocytometer.* Mycoplasma* contamination was tested regularly with PCR LookOut kit (Sigma).

For the restricted nutrient condition and stable isotope–labeling experiments, cells were cultured using DMEM (no glucose, no glutamine, no phenol red, Thermo Fisher Scientific, #A1443001) complemented with glucose, glutamine, and pyruvate, as indicated. For the stable isotope labeling, the required amount of labeled compounds, [U-^13^C] glucose, [U-^13^C] glutamine, and [U-^13^C] pyruvate, were added. FBS was dialyzed using a SnakeSkin dialysis tubing (Thermo Fisher Scientific) and filtered using 0.22 µm PES filter before being added in 10% proportion, together with Normocin. The conditions were named according to their nutrient concentrations: high and low glutamine (H/L = 1/0.1 mmol/L) levels were combined with the presence and absence of pyruvate (+/− = 1/0 mmol/L), glucose was kept at 10 mmol/L.

### Discovery of large gene cluster switches with MCbiclust

MCbiclust [doi:10.18129/B9.bioc.MCbiclust, current version 1.2.1, R package available in Bioconductor (22)] was used to identify gene cluster switches as described previously (21, 23).

The method is designed to find subsets of samples in which large gene sets, such as sets of metabolic and nuclear encoded mitochondrial genes (23, 24), are coregulated, as demonstrated by the correlation of individual gene expression levels across the selected samples. Technically, an iterative stochastic greedy search method selects the group of samples in which the chosen gene set reaches the maximum absolute correlation score. The samples and gene sets that have the highest score define a bicluster.

**Figure 1.**
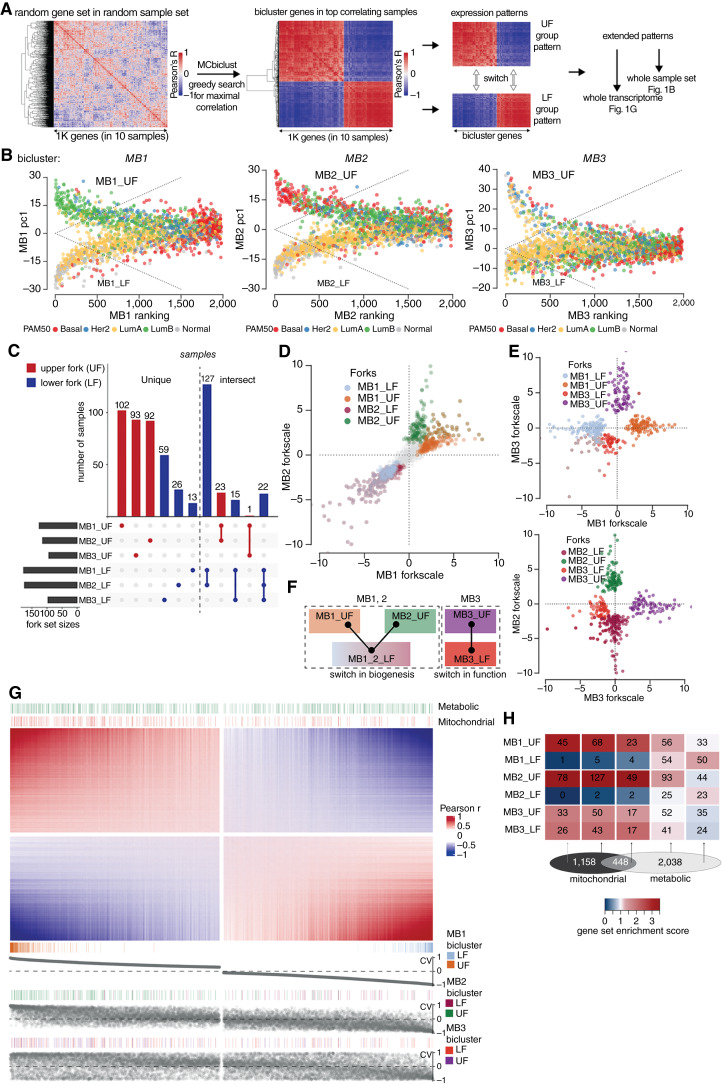
Large-scale transcriptome switches identify breast cancer subtypes. **A,** Gene set switch identification in bicluster samples. MCbiclust starts with a random gene set in a random sample set and performs a greedy search aiming for the sample set, across which, the gene set has the highest correlation. As the absolute value of correlation is maximized during the search, the resulting top correlating samples display two gene sets with sharp anticorrelation (switch) between them. The gene sets and sample sets can be extended to the whole transcriptome and sample set by quantifying correlations (see main text), which provides the basis for gene set enrichment analysis. **B,** Visualization of individual switches (MB1, MB2, MB3 biclusters) in all METABRIC samples. Two-dimensional distribution by PC1 and ranking index are shown, overlaid with PAM50 classifiers. Dashed lines represent the thresholds to include samples in the forks, calculated as 0.04 × forkscale (see Materials and Methods). **C,** UpSet matrix and plots of sample intersections between top and bottom positions of each bicluster switch. Unique and overlapping (intersect) samples are shown separately. **D,** Scheme of switches. MB1 and MB2 share their LF, thus representing a multistate switch, while MB3 is an independent bistate switch, thus the MB1_2 and MB_3 biclusters represent different switch mechanisms. **E** and **F,** Interswitch relationships are shown in two-dimensional distribution plots of samples by two biclusters (switches). The axes represent the scale between the UF and LF of each bicluster. The distribution of samples according to their MB1 vs. MB2 forkscale value (see Materials and Methods) is shown in **E**, while the combinations of MB3 with MB1 or MB2 are shown in **F**. **G,** Heatmap of the correlation values (CVs, Pearson *r*) across the whole transcriptome in the MB1 bicluster; a small set of midrange values were hidden (see gaps in heatmap). Annotations show the locations of mitochondrial and metabolic genes (top), and bicluster genes (bottom). For comparison between the switches, bottom annotation plots show the CV values of each sample in the three biclusters (switches). **H,** Fold enrichment of mitochondrial and metabolic (with intersections) genes in the anticorrelated gene sets. Heatmap shows enrichment values of each gene set in each position of all switches. Gene set intersections are on the Venn diagram below the heatmap.

As an additional feature, MCbiclust uses the absolute correlation values for sample selection, resulting in two gene groups with maximal positive correlations within the groups but negative correlation between each other. Thus, two gene sets with mutually exclusive expression are obtained in each bicluster, defining two groups of samples (23). Importantly, after defining the bicluster, quantitative correlation is extended to the (i) whole sample set using the similarity of each sample to the bicluster sample set, ranking them according to the correlation of their bicluster gene set expression with that of the bicluster samples, and (ii) to the whole transcriptome, using the correlation of each gene transcript with the average expression of a set of representative bicluster genes (correlation vector values, CV). Thus samples and gene sets can be analyzed independently (for the schematics of the method see Fig. 1A).

Here we applied the method to the METABRIC (25), The Cancer Genome Atlas (TCGA)-BRCA (26), and Oslo2 (27) datasets. We have used two defined and two random starting gene sets of approximately 1000 genes. The defined gene sets were either only mitochondrial (Mitocarta 1.0; ref. 28) or mitochondria related, by choosing the 1,000 genes most correlated with the mitochondrial ribosomal gene MRPL58 across the METABRIC dataset. MCbiclust was run for 1,000 iterations using each gene set on a University College London high performance cluster. The silhouette method was used to determine the number of independent biclusters. Biclusters were then extended (i) by calculating the correlation vector (CV) values for the whole transcriptome using the CVEval function and (ii) to all samples using SampleSort. The correlation pattern was summarized using principal component analysis of the CV values using PC1VecFun. The bifurcating patterns, defining the forks (switch positions), were plotted using the sort order (index) and PC1 values for each sample.

### Metabolic phenotype prediction from transcriptomic data

For gene group discovery, we selected groups of genes with maximum correlation. This reflects strong coexpression/coregulation among the genes in the group. For the selection of the gene group members we maximized the absolute correlation value across the group, thus we considered both negative and positive correlations. Correlation was calculated for the whole transcriptome and used to quantify the correlation of each transcript in a metabolic pathway with the prevailing pattern, giving a ranked distribution of the pathway members associating with the GEP, primarily indicating whether the correlation values are higher in the upper fork (UF) or lower fork (LF) groups. This difference usually coincides with the difference in overall abundance of transcripts in a pathway. Thus, we predicted relative differences in pathway activities between two groups of samples (UF and LF) in a bicluster, based on overall transcriptional activity and regulation in the groups along the specific pathway.

### Pathway analysis and cell line scoring

Signaling pathway activity was assessed using the PARADIGM integrated pathway levels (IPL) from the PARADIGM (29) analysis on a pan-cancer dataset (https://www.synapse.org/#!Synapse:syn5633407; ref. 30), filtered for BRCA samples. Gene set variation analysis (GSVA) for molecular signature identification was performed using the GSVA 1.46.0 package (31). For sorting breast cancer cell lines in bicluster groups, we used GSVA scoring on breast cancer cell line transcriptome dataset from CCLE (32), ComplexHeatmap was used for visualization (33).

### Assessment of mitochondrial activity

Oxygen consumption rate was measured with the Seahorse XFe96 bioanalyzer using the Seahorse XF Cell Mito Stress Test Kit (Agilent) or using High-Resolution Respirometry (Oxygraph-2K, Oroboros Instruments). For the Seahorse measurements, cells were seeded on XF96 cell culture microplates (Agilent) 2 days before the experiment (1 × 10^4^ cells/well). For the experiment, the culture medium was replaced with Seahorse XF Base medium (Agilent) supplemented with pyruvate, glutamine, and glucose (Thermo Fisher Scientific) as indicated, and incubated for 30 minutes at 37°C in a CO_2_-free incubator before loading into the Seahorse analyzer. After measuring basal respiration, the drugs oligomycin A (5 µmol/L), carbonyl cyanide-p-trifluoromethoxyphenylhydrazone (FCCP;1 µmol/L), and rotenone/antimycin A (0.5/0.5 µmol/L) were added to each well in sequential order. Data were analyzed using the XF Cell Mito Stress Test Report Generator. After the assay, cells were stained with Hoechst 33342 (1 µg/mL; Thermo Fisher Scientific) for 30 minutes. ImageXpress Micro XL was then used for cell nuclei counts in each well for normalization to cell numbers.

For High-Resolution Respirometry the Oxygraph-2K instrument was used. Prior to the assay, OROBOROS chambers were calibrated with the recording media [DMEM, #A1443001, glucose (10 mmol/L), glutamine (1 mmol/L), pyruvate (1mmol/L), HEPES (10 mmol/L)]. Confluent 10 cm plates of cells were trypsinized and resuspended in the same media. A total of 2.5 × 10^6^ cells were added to each chamber, and the O_2_ flow signal was allowed to stabilize to basal respiration rate. Drugs were added to chambers using the following concentrations and order: oligomycin (2.5 µmol/L), FCCP (2 µmol/L), and antimycin A (2.5 µmol/L). Respiratory rate given as oxygen flow in pmol/minutes/cell was recorded. Data collection and analysis was performed using Datlab 5.0 software.

### Imaging

For imaging overall mitochondrial structure and function in a large cell population, a wide-field high content imaging system (ImageXpress MicroXL, Molecular Devices) was used. Cells were seeded in 96-well black clear thin bottom tissue culture treated imaging plates (Corning). For mitochondrial membrane potential measurements, cells were incubated with tetramethylrhodamine methyl ester (TMRM, 30 nmol/L; Thermo Fisher Scientific) in recording media [DMEM, #A1443001, glucose (10 mmol/L), glutamine (1 mmol/L), pyruvate (1 mmol/L), HEPES (10 mmol/L)] for 30 minutes prior to starting imaging and left for the whole time of the experiment at 37°C. For labeling the nuclei, cells were coloaded with Hoechst 33342 (1 µg/mL; Thermo Fisher Scientific) for 30 minutes. For TMRM intensity quantification a 20× Nikon (S PLAN FLUOR ELWD 0.45 NA) air objective was used. Images were analyzed with the integrated metaXpress imaging and analysis software using the granularity analysis module. Following local background subtraction, the average TMRM intensity/field was used as readout.

For morphology analysis, cells were coloaded with TMRM (30 nmol/L), picoGreen (2.5 µL/mL) and Hoechst 33342 (1 µg/mL; Thermo Fisher Scientific) in recording media [DMEM, #A1443001, glucose (10 mmol/L), glutamine (1 mmol/L), pyruvate (1 mmol/L), HEPES (10 mmol/L)] for 30 minutes prior to starting imaging. TMRM was left for the whole time of the experiment at 37°C. Cells were imaged using a custom protocol to image three wavelengths [Lumencor solid state illumination with Semrock (Brightline) filters (nm) ex377/50 em447/60 for Hoechst 3342; ex472/30 em520/35 nm for picoGreen, ex531/40 em593/40 for TMRM], 16 fields/well with a Nikon 40× Plan Apo NA 0.95 air objective, binning 1 with a CMOS detector. TMRM and picoGreen images were analyzed with MetaXpress 5.0 software, using the granularity module, total mitochondrial area, and average object size as readout from the TMRM images. For picoGreen analysis, the integrated pit intensity (granularity module) was used as a readout following ratioing with the Hoechst images, background subtraction, and thresholding.

### Determination of total cellular ATP

Total cellular ATP concentration was measured using CellTiter-Glo Luminescent Cell Viability assay (Promega). The method determines the number of viable cells based on the quantification of the ATP present, as readout of metabolically active cells, thus allowing the estimation of total cellular ATP produced by a known number of seeded cells. In detail, 5K cells were seeded in an opaque black 96-well plate and treated with the experimental media for 24 hours. Wells containing the experimental medium only (the blank—for removing the medium background) and 10 nmol/L ATP standard (to which the samples’ luminescence was normalized to) were included. The protocol was carried out according to the manufacturer’s instructions. Briefly, (i) plates were equilibrated at room temperature for approximately 30 minutes, avoiding temperature gradients that could cause uneven signals; (ii) a volume of CellTiter-Glo Reagent equal to the volume of cell culture medium present in each well (e.g., 100 μL of reagent to 100 μL of medium containing cells for a 96-well plate) was added; (iii) 2 minutes of orbital shaking favoring cell lysis; then, (iv) plates were left at room temperature for 10 minutes to stabilize luminescent signal; and (v) the luminescence was recorded using PerkinElmer EnVision plate reader. For each plate the triplicate values for the samples total ATP produced, the ATP standard and five replicates of medium luminescence for the background were acquired. The average of the replicates was calculated, and the medium background was subtracted. The resulting values were normalized to the ATP standard previously adjusted for the background and further normalized to the protein content, evaluated using the BCA protein assay (Pierce).

### Western blotting

For the baseline expression of metabolic enzymes and mitochondrial respiratory chain subunits at protein level, proteins were extracted from cells plated in 10 cm dish and cultured in DMEM. Briefly, cells were detached by scraping in cold PBS 1× and pulled down by centrifugation. Whole cell lysate was obtained incubating cell pellet with cold RIPA buffer [150 mmol/L NaCl, 50 mmol/L Tris, 0.5% sodium deoxycholate, 0.1% (w/v ) SDS, 1% (v/v) Triton] in presence of protease inhibitor cOmplete (Roche), phosphatase inhibitors PhosStop (Roche), and PMSF (Sigma) for 30 minutes on ice and spun down at 4°C. Protein quantification was done using a BCA protein assay kit (Pierce). Samples were denatured in presence of reducing agents (DTT) at 95°C for 5 minutes, while for probing mitochondrial respiratory chain subunits the optimal temperature was 60°C for 10 minutes. 20 µg of protein lysate was loaded onto a NuPAGE 4% to 12% bis-tris gel (Thermo Fisher Scientific) and run using MOPS 1× buffer. Blotting was done for 2 hours at 30 V in a wet system (Invitrogen; Thermo Fisher Scientific), by transferring proteins onto nitrocellulose membrane for metabolic enzymes and PVDF for mitochondrial subunits. Membranes were incubated in 5% non-fat dry milk in TBS 1×–0.1% Tween 20 or 5% BSA in TBS 1×–0.1% Tween 20 as blocking buffers (according to each antibody datasheet) for 1 hour at room temperature. Primary antibody was incubated in blocking buffer overnight at 4°C. The working dilution was 1:1,000 for the most of antibodies except for MitoProfile (1:2,000), grp75 (1:3,000), actin (1:3,000). Horseradish-conjugated secondary antibodies (Bio-Rad) were diluted 1:5,000 in blocking buffer and membranes were incubated 1 hour at room temperature. The blots were visualized using SuperSignal West Pico Chemiluminescent Substrate (Thermo Fisher Scientific) and UVITEC Cambridge Mini HD9 Imaging System (Eppendorf). If necessary, membranes were stripped, blocked, and probed again with a different primary antibody. Signal intensities of specific bands were quantified with Fiji (34).

### Measurement of glutamine and glutamate concentration in the media

The concentration of glutamine and glutamate in the media were measured using the luminescence-based approach Glutamine/Glutamate-Glo Assay kit (Promega). The method is based on two steps: (i) glutamine is first converted to glutamate by glutaminase; (ii) glutamate is oxidized and NADH produced is used for luminescence reading. Briefly, 10k cells were seeded in an opaque black 96-well plate. On the following day, after one wash with warm PBS 1×, cells were treated with the experimental media for 24 hours. Wells containing the experimental medium only (no cells), five points glutamate standard curve (to extrapolate glutamine and glutamate concentration), and wells containing PBS 1× (as negative control) were included. Wells with only medium represent a positive control and were used as reference of initial glutamine and glutamate concentrations in the medium, from which glutamine and glutamate concentration values obtained from cell-containing wells were subtracted, thus calculating the glutamine uptake and glutamate secretion by the cells. The addition and removal of both PBS 1× and media were gently done using a multichannel pipette. Each experimental medium was freshly prepared on the day of the treatment. The experiments were carried out according to the manufacturers’ instructions. After 24 hours treatment, media were collected and a dilution of 1:25 (in PBS 1×) was done in order to fit the linear range of the assay indicated by the commercial kit. The diluted samples were analyzed the same day or frozen at −80°C and processed afterwards. The assay was done at room temperature with media and reagent RT equilibrated to avoid uneven signals. Luminescence was recorded using Infinite M200 plate reader (Tecan). Protein content was evaluated using the BCA protein assay (Pierce). The results were presented as nmoles/minutes/grams of protein.

### Stable isotope labeling, metabolite extraction, and quantification

In order to detect the metabolic switch with altered substrate preferences associated with the different mitochondrial switch positions, we performed a steady state metabolic flux analysis of heavy carbon–labeled key cellular fuels using uniformly labeled ^13^C-glucose, ^13^C-glutamine, and ^13^C-pyruvate. MB1_UF and MB1_LF cells were incubated with each substrate in separate experimental sets. Mass spectrometry analysis of carbon-labeled compounds was used to quantify isotopologues of metabolites in the main catabolic pathways of the three substrates. Cells were grown at standard culturing conditions as described in cell culture methods; the stable-isotope labeling was obtained by culturing cells with media containing either fully labeled [U-^13^C] glucose, [U-^13^C] glutamine, or [U-^13^C] pyruvate for 24 hours. Before metabolite extraction, plates were taken to a cold room and 500 μL of medium from each plate was frozen for later analysis. The remaining media was removed, and the plates placed in Fan ice/water bath before washing twice with ice-cold PBS.

For metabolites extraction, 800 μL ice-cold methanol containing an internal standard of 1 mmol/L scyllo-inositol was added to each plate and cells were detached by scraping. This mixture was transferred to a 15 mL tube, and the plate washed with 800 μL of methanol:H_2_O (1:1 vol/vol) that was moved to the tube, to which 400 μL of ice-cold chloroform was added. The tubes were placed in a water bath sonicator in a cold room for 1 hour, with 3 × 8 minutes pulses of sonication and centrifuged for 10 minutes at 16,000 rpm at a temperature of 0°C. The supernatant was extracted and dried in a vacuum concentrator. The cell pellet was then re-extracted with 300 μL of methanol:H_2_O (2:1 vol/vol), this was sonicated, spun, and the supernatant added to the previous supernatant tube and dried again in a vacuum concentrator. The remaining cell pellet was used for estimating dry weight and measuring total protein. For phase partitioning, the dried supernatant was resuspended in 350 μL of chloroform:methanol:H_2_O (1:3:3 vol/vol) and spun for 5 minutes at 0°C and 16,000 rpm. The extract is then in a biphasic partition, with the upper phase containing the polar metabolites and the lower phase containing lipidic metabolites. The polar phase fractions were then transferred to GC-MS vial inserts and vacuum dried. Separate vial inserts had 10 μL of the saved cell culture medium added, with 1 mmol/L scyllo-inositol, which were also dried in a vacuum concentrator. Each vial insert had 30 μL of methanol added, containing 1 μL of 5 mmol/L nor-leucine as another internal standard, followed by 30 μL of methanol without nor-leucine, with the vials being dried in a vacuum concentrator after each addition. Before running samples on the mass spectrometer, polar fractions were derivatized, 20 μL methoxyamine (30 mg/mL in pyridine) was added to each insert and this was vortexed briefly and incubated at room temperature overnight, silylation was then done by adding 20 μL of BSTFA + TCMS reagent to each insert and incubating for 1 hour at room temperature.

For metabolite analysis, an Agilent 7890A GC with a 5975C triple axis detector MSD (Agilent Technologies) was used. Metabolites were separated on an Agilent J&W 122-5532G DB-5 ms capillary column (30 m × 0.25 mm, 0.25 µm film thickness), in splitless mode. The injector and transfer line temperatures were 270°C and 280°C, respectively. The flow rate of helium carrier gas was 0.7 mL/minutes. The oven temperature was programmed to hold at 70°C for 2 minutes, increased to 295°C at a 12.5°C/minutes ramp rate, increased from 295°C to 320°C at a 25°C/minutes ramp rate, and held at 320°C for 3 minutes. The mass spectrometer was operated in scan mode, after a 6 minutes solvent delay with a range of 50–565 mass/charge (*m*/*z*) and a scan rate of 2.8 scans per second. Metabolites were identified by matching retention times and fragmentation patterns to commercially available standards. Metabolite peaks were integrated at each isotopologue *m*/*z* using MassHunter Workstation software (Agilent Technologies). Peak areas were quantified based on peak areas of known standards using nor-leucine as an internal standard, and then metabolite levels were normalized to protein content. Mass isotopologues were stripped of the contribution from natural abundance, based on the chemical formula of derivatized fragments quantified. Percent enrichment for an isotopologue was calculated by dividing the corrected intensity by the sum of corrected intensities of all isotopologues for that metabolite. Significance of metabolite enrichment between different samples was calculated with one-way ANOVA.

### Data availability

The METABRIC transcriptomic dataset analyzed in this study was obtained from Synapse at syn1757063 (https://doi.org/10.7303/syn1688369). The TCGA transcriptomic dataset analyzed in this study was obtained from NCBI dbGaP at phs000178.v11.p8. The OsloVal transcriptomic dataset analyzed in this study was obtained from Synapse at syn1710395 (https://doi.org/10.7303/syn1688370). All other raw data generated in this study are available in the manuscript and supplementary tables and upon request from the corresponding author. The code and data files used for analysis and creating the figure are available on GitHub (https://github.com/gszabadkai/Menegollo_Bentham).

## Results

### Robust gene cluster switches define mitochondrial and metabolic subtypes of breast cancer

In order to identify coregulated gene clusters in subsets of breast cancer samples, we used a massive correlating biclustering method, MCbiclust, developed to classify large transcriptome datasets based on correlated gene expression modules (Fig. 1A; ref. 22). MCbiclust identified three independent gene-sample biclusters in the METABRIC dataset (termed mitochondrial biclusters MB1, MB2, and MB3; Supplementary Fig. S1A–S1C), revealing three large, robustly coregulated gene sets in specific subsets of the METABRIC samples (31). The gene sets were composed of two distinctly anticorrelated gene groups (Supplementary Table S1). When samples were ranked by the average absolute correlation strength of their bicluster genes (correlation value, CV), they showed a fork-like distribution according to the expression values (PC1) of the two anticorrelated gene groups. Thus, each sample in the biclusters is sorted to either position of a toggle switch, the UF and LF groups (Fig. 1B). The characteristic distribution pattern of the samples encapsulated the split nature of the biclusters, with bifurcating PC1 values in highly ranked samples. The biclusters and toggle switches were confirmed in the TCGA (32) and Oslo2 (33) breast cancer cohorts (Supplementary Fig. S1D–S1F).

While most forks of the three biclusters were composed of unique samples, MB1_LF and MB2_LF showed a near complete overlap and were therefore aggregated into a common cluster, MB12_LF (Fig. 1C and D). To compare the position of the samples in all discovered biclusters, we used their “forkscale” value, the position relative to the tip of the upper (+1) or lower (−1) bicluster forks (Fig. 1E and F). The MB1 and MB2 bicluster switches showed tripolar distribution where samples are merged in a common LF (MB12_LF) and are split between two distinct UFs, MB1_UF and MB2_UF. The tripolar distribution indicated a multistate switch. Comparison of MB3 with either MB1 or MB2 did not show any significant overlap, indicating independently regulated gene cohorts in the MB3 bicluster (Fig. 1F).

To define the gene sets dominating the biclusters we have ranked the whole transcriptome based on the correlation of each gene with the bicluster genes (CV). The correlation map of the MB1 bicluster is shown in Fig. 1G. Genes with the highest and lowest CVs were considered the dominant, most strongly regulated genes in the UF and LF switch positions, respectively. Importantly, genes with the highest CVs (in both UF and LF switch directions) include genes additional to the bicluster (Supplementary Table S1), indicating that they are part of larger scale gene regulation patterns. Notably, nuclear encoded mitochondrial and metabolic genes were markedly over-represented in the UF of the MB1 and MB2 bicluster patterns but were excluded from the MB12_LF. The relative enrichment of nuclear encoded mitochondrial and metabolic genes is shown in Fig. 1H.

Altogether, MCbiclust identified large-scale gene expression switches defining breast cancer subtypes, involving robust coregulation of a large group of metabolic and mitochondrial genes.

### Metabolic and mitochondrial transcriptional clusters extend over breast cancer subtypes and associate with cell of origin

Mapping all metabolic genes of the three biclusters on the global Kyoto Encyclopedia of Genes and Genomes (KEGG) metabolic pathway map (Supplementary Fig. S2A) indicated that the transcriptional switches comprise all major metabolic modules. Switches were represented by alternating dominance of mutually exclusive pathways (e.g., lipid synthesis versus degradation) or enzyme isotypes.

**Figure 2.**
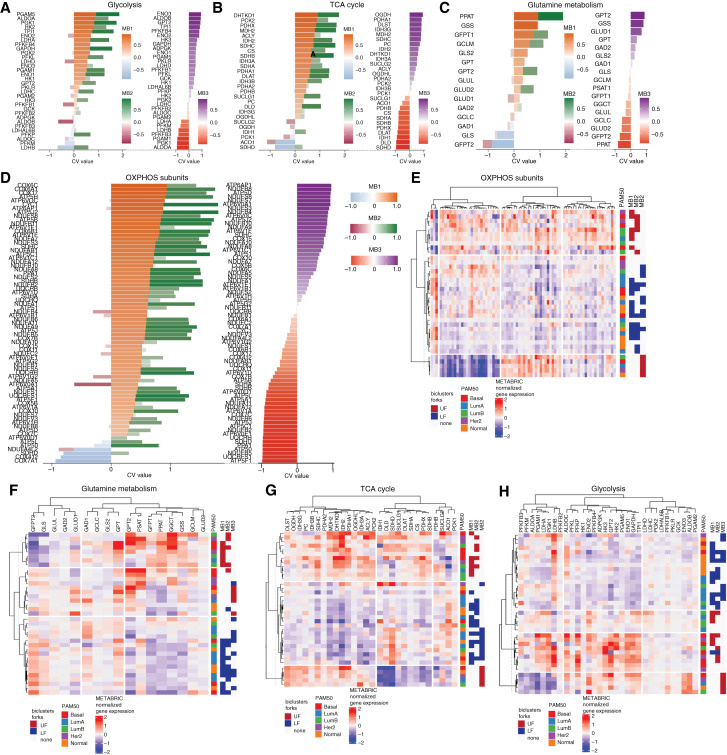
Metabolic transcriptome switches override PAM50 classification. **A–D,** Visualization of bicluster switches based on CV values of individual genes in the METABRIC breast cancer dataset analysis in the indicated metabolic pathways. Genes are ranked by the MB1 CV values and directly compare the MB1 and MB2 biclusters on the left side of each panel. The ranked MB3 values are shown on the right side of each panel. **E–H,** Sub-pathway gene clusters group according to the bicluster switches across PAM50 categories. Bicluster forks samples were grouped according to their intrinsic subtype and the average transcript levels of each gene in the indicated pathways were calculated. Hierarchical clustering of genes and sample groups was performed (Pearson distance and ward2 clustering). Group classifications according to the biclusters and intrinsic breast cancer subtypes are shown on the right.

To predict differential metabolic wiring in central carbon metabolism, first we compared the CVs of each gene in individual metabolic pathways, in different switch positions and biclusters (Fig. 2A–D; Supplementary Fig. S2B). The majority of genes in the mitochondrial and coupled metabolic pathways (KEGG: oxidative phosphorylation, TCA cycle, glycolysis, glutamine, and pyruvate metabolism; Mitocarta: mitochondrial central dogma) showed strong positive correlation with the UF switch positions in the MB1 and MB2 biclusters, while contrasting gene clusters were found in the MB3 bicluster. To quantify the effect of coregulation on individual transcripts, next we compared the average normalized expression levels of each gene in the METABRIC dataset in the switch positions of all biclusters. As metabolic differences between intrinsic subtypes have been contended (35) and switch positions in the MB1 and MB2 biclusters partially overlap with the PAM50 classification (Supplementary Fig. S2C), we further divided the bicluster sample groups by their intrinsic subtype and applied hierarchical clustering to understand the key determinants of the differences (Fig. 2E–H). Genes in all pathways primarily clustered by their switch position. The MB1 and MB2 gene groups showed significant overlap but the MB3_UF group clustered separately. Importantly, the clusters comprised all PAM50 subtypes, indicating that MCbiclust is able to find coregulation across, and independently of, the intrinsic subtypes. Overall, the metabolic switch in the MB1 and MB2 biclusters encompassed increased mitochondrial biogenesis and predicted higher electron flux through OXPHOS compared to MB12_LF. On the other hand, glycolysis, pyruvate metabolism, and glutaminolysis, feeding into the TCA cycle, indicated a change of transcriptional pattern in distinct switch positions, suggesting altered substrate preference. While TCA cycle genes involved in pyruvate oxidation dominated MB1 and MB2 UFs, MB12_LF showed high GLS/PPAT ratio, indicating glutamine usage for OXPHOS (36).

**Figure 3.**
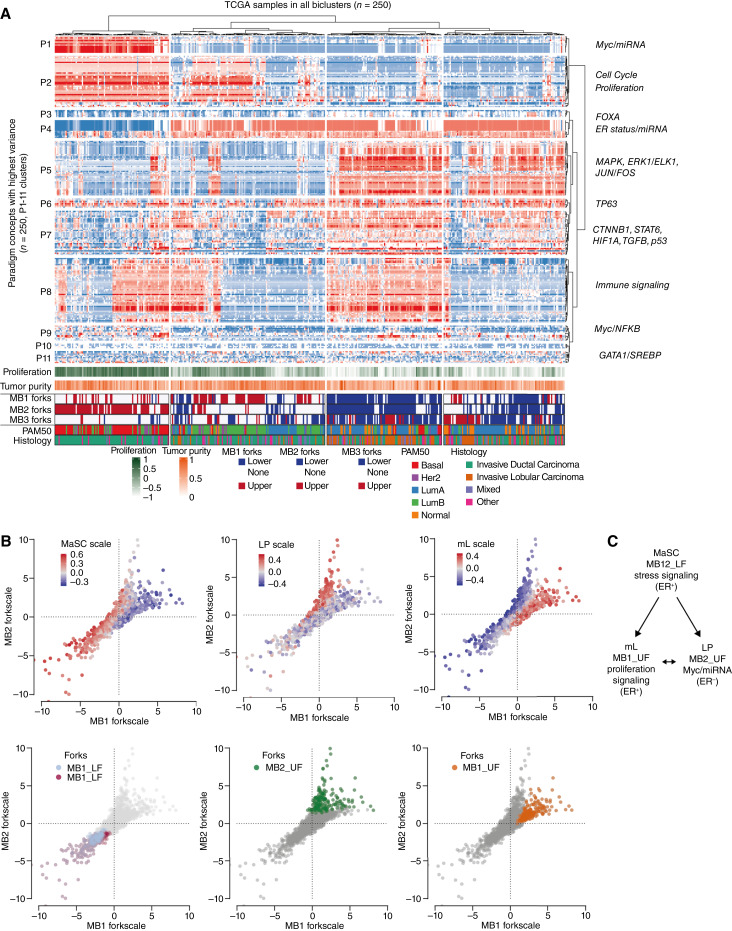
Oncogenic signaling and cell of origin associates with the biclusters. **A,** Heatmap of distribution of the top 250 most variable PARADIGM concepts across the bicluster switches in the TCGA-BRCA dataset. The term values were obtained from Berger and colleagues (30) pan-cancer analysis and hierarchically clustered. Annotations show proliferation, tumor purity, switch identity (UF and LF of MB_1, MB_2, or MB_3), Pam50, and histology classifications. Transcriptomic signatures based on PARADIGM concepts are shown on the right. **B,** Association of mammary gland cell states (37) and transcriptomic switches in the METABRIC dataset. Two-dimensional distribution plots of samples by two biclusters are shown overlaid with cell-of-origin marker GSVA values. **C,** Scheme of signaling of the multistate switch in the MB1 and MB3 biclusters.

Next, to infer signaling pathways associated with the metabolic transcriptional signature, we analyzed the distribution of PARADIGM activities (29) on a pan-cancer dataset (30) among different switches (Fig. 3A). The switch between lower and UF positions is dominated by turning on a cell proliferation cluster (MYB, FOXM1, E2F family, *P2*) in the UFs (MB1_UF, MB2_UF) and by a stress and hypoxia (MAPK, p53, HIF1, *P5-7*) and stemness (p63) signaling cluster in the LFs (MB12_LF). Interestingly, the MB1_UF and MB2_UF samples clustered separately, likely due to activation of Myc in MB2_UF together with a miRNA pattern (*P1*). While both MB1_UF and MB12_LF switch positions showed strong estrogen receptor signaling (*P4*), MB2_UF contained only ER negative samples (PARADIGM concept lists in Supplementary Table S2; statistical analysis in Supplementary Fig. S3). All bicluster switches contained only a fraction of samples with high immune signature, indicating that differences between the forks is not determined by the immune system.

Finally, we assessed whether the identified metabolic and signaling switches among groups of breast cancer samples relate to their cell of origin (37). The GSVA scores (31) for gene expression patterns derived from mammary stem cells (MaSC), luminal progenitors (LP), and mature luminal (mL) cells were overlaid on the fork distribution plots, showing association between the three cell states and the MB12_LF/MB1_UF/MB2_UF multistate switch (Fig. 3B; Supplementary Fig. S4A). Average scores of all three cell states were significantly higher in the bicluster groups than for any of the intrinsic (PAM50) subtypes (Supplementary Fig. S4B). Overall, stemness (MaSC) was largely accompanied by MB12_LF features, such as low mitochondrial abundance and glutamate substrate preference, while switching to either mL or LP cell state was followed by increased mitochondrial biogenesis driven by proliferation signaling in absence (MB1_UF: mL state) or presence (MB2_UF: LP state) of Myc/miRNA signals (Fig. 3C).

### Mitochondrial and metabolic gene expression profiles predict functional metabolic switches

**Figure 4.**
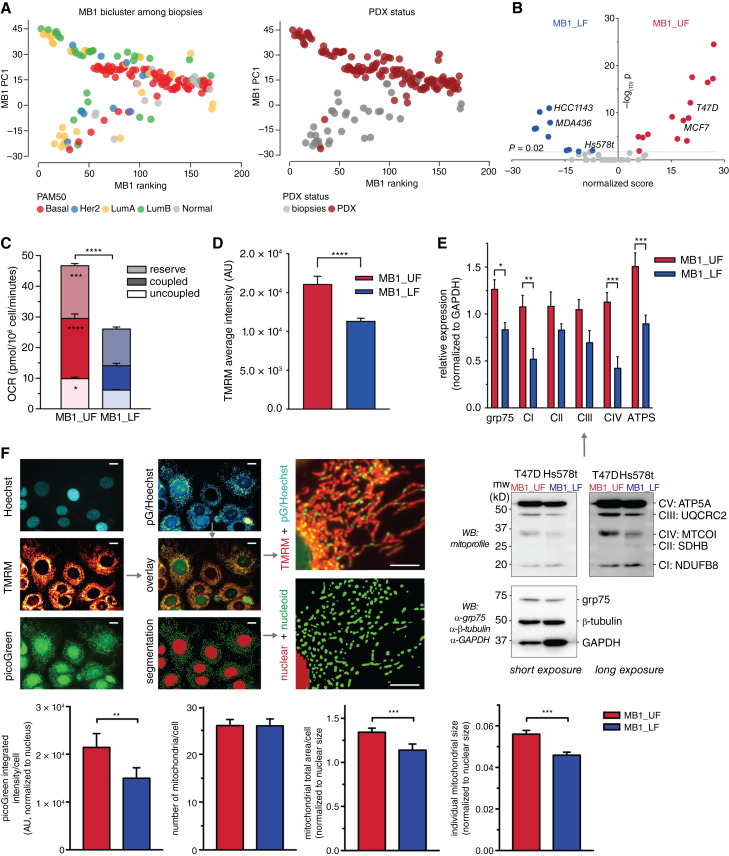
Mitochondrial function: MB1_UF cells show active functional biogenesis in contrast to the MB1_LF phenotype. **A,** Two-dimensional distribution (PC1 vs. ranking index, shown for the MB1 bicluster switch) of biopsies obtained for generating PDX samples from the study by Bruna and colleagues (38), overlaid in left panel with Pam50 categories and in the right panel with PDX status indicating whether the sample has grown from the biopsy to PDX status. **B,** Cell line scoring to sort cell lines to the MB1 switches. Volcano plot of the scores and −log_10_ of the *P* values are shown. The cell lines chosen for experimental work are highlighted in red. **C,** OCR in cell lines grouped by their switch state (MB1_UF/MB1_LF). Reserve, ATP synthesis coupled, and uncoupled respirations were quantified using FCCP and oligomycin treatments. Two-way ANOVA with Sidak multiple comparisons test. **D,** Average cellular TMRM intensity grouped by cell line switch state (MB1_UF/MB1_LF). Data were obtained from high content microscopy from >1K cells per group. Unpaired Student *t* test. **E,** Relative expression of respiratory chain subunits quantified from Western blot analysis. Results are grouped by switch state (MB1_UF/MB1_LF). Two-way ANOVA with Sidak multiple comparisons test. **F,** High content imaging analysis of mitochondrial biogenesis, structure, and function in MB1_UF and MB1_LF cells. Scale bars, 10 μm. Quantifications of the picoGreen integrated intensity (readout for mtDNA content), mitochondrial number, total volume, and individual mitochondrial volume are shown. Unpaired Student *t* tests. ns, nonsignificant, *P* > 0.05; *, *P* ≤ 0.05; **, *P* ≤ 0.01; ***, *P* ≤ 0.001; ****, *P* ≤ 0.0001.

For functional verification of the predicted metabolic switches, we tested the hypothesis whether cells with MB1_UF phenotype (i) boast higher mitochondrial content and activity and (ii) have altered substrate preferences as compared with the MB1_LF phenotype. In a collection of patient-derived breast cancer xenograft models (38), the MB12_LF position was not represented among the successfully implanted PDXs, although all the biclusters were identified in the primary tumors (Fig. 4A). Thus, using a custom scoring method, we identified breast cancer cell lines representing each position of the three bicluster switches (MB1_UF: MCF7, T47D; MB1_LF: MDA-MB-436, Hs578t, HCC1134; Fig. 4B; Supplementary Table S3).

MB1_UF cells had higher oxygen consumption rate and mitochondrial membrane potential, indicating higher redox potential to feed electrons in the respiratory chain (Fig. 4C and D). Higher mitochondrial abundance in MB1_UF cells was confirmed by Western blot analysis of respiratory chain components (Fig. 4E). High-resolution image analysis of mitochondria across cell populations (Fig. 4F) showed that MB1_UF cells had higher mtDNA content and greater total and individual mitochondrial area, indicating larger mitochondrial size and overall content. These experiments confirmed a switch in mitochondrial abundance and function as predicted by gene expression profiling using MCbiclust.

**Figure 5.**
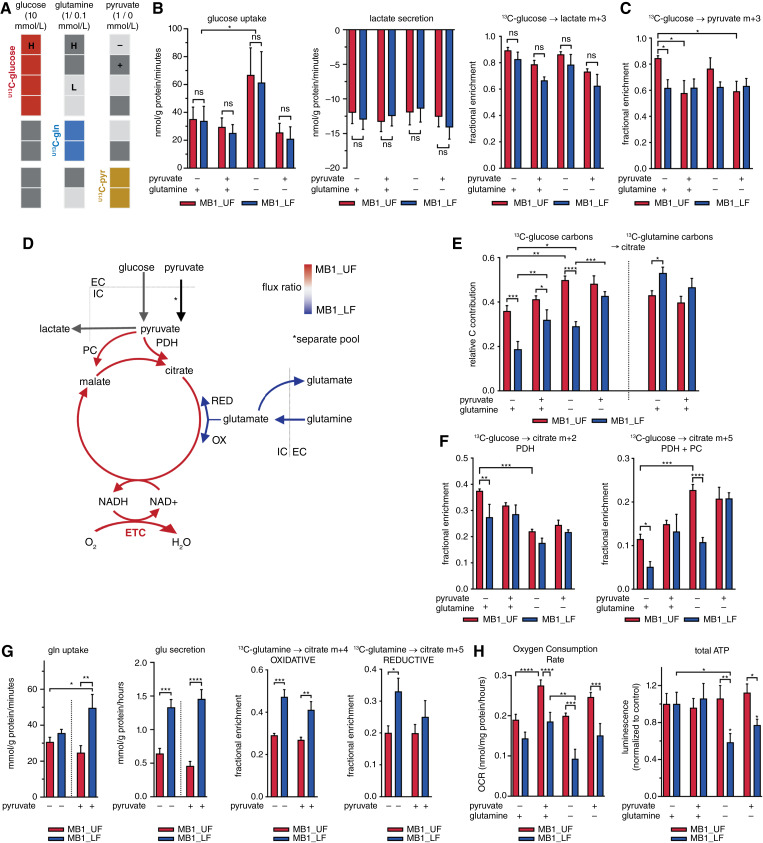
Metabolic wiring of the switch positions probed by ^13^C-labeled substrates. **A,** Nutrient supply and ^13^C-labeling conditions. High and low glutamine (H/L = 1/0.1 mmol/L) levels were combined with the presence and absence of pyruvate (+/−, 1/0 mmol/L), and glucose was kept at 10 mmol/L. **B,** Glucose uptake, lactate secretion, and fractional enrichment of m + 3 labeled intracellular lactate. Cells were grouped by switch state. Two-way ANOVA with Sidak multiple comparisons test. **C,** Fractional enrichment of the m+3 pyruvate isotopomer, following ^U13^C-glucose labeling for 24 hours, in cells grouped by switch state. Two-way ANOVA with Sidak multiple comparison tests. **D,** Overall model of the MB1_UF/MB1_LF metabolic switch in central carbon metabolism. **E,** Relative carbon contribution to citrate following 24 hours labeling with ^U13^C-glucose and ^U13^C-glutamine, in cells grouped by switch state. Two-way ANOVA with Sidak multiple comparison tests. **F,** Fractional enrichment of the m+2 (PDH route; left) and m+5 (PDH + PC route; right) isotopologues in citrate following 24 hours labeling with ^U13^C-glucose, in cells grouped by switch state. Two-way ANOVA with uncorrected Fisher LSD multiple comparison tests. **G,** Glutamine uptake, glutamate secretion, and fractional enrichment of malate m+4 (oxidative route) and citrate m+5 (reductive carboxylation) isotopologues following 24 hours labeling with ^U13^C- glutamine (right panels). Two-way ANOVA with uncorrected Fisher LSD multiple comparison tests. **H,** Substrate dependence of basal oxygen consumption rate and total cellular ATP content in cells grouped by switch state. Two-way ANOVA with uncorrected Fisher LSD multiple comparison tests. ns, nonsignificant, *P* > 0.05; *, *P* ≤ 0.05; **, *P* ≤ 0.01; ***, *P* ≤ 0.00; ****, *P* ≤ 0.0001.

No differences were found between the MB1_UF and MB1_LF switches in glucose uptake, lactate production, and the relative incorporation of glucose-derived heavy carbons into lactate (Fig. 5A and B). Thus, aerobic glycolysis occurs in both MB1 switches at high rate, resulting in nearly complete glucose-derived ^13^C-labeling of glycolytic intermediates (Supplementary Fig. S5A). However, MB1_UF cells produced more pyruvate (m+3) from glucose than MB1_LF cells (Fig. 5C), suggesting that glucose flux through glycolysis was higher in the MB1_UF switch, delivering pyruvate oxidized in the TCA cycle.

To assess substrate preferences (Fig. 5D), we quantified the relative carbon contribution of glucose, glutamine, and external pyruvate to the TCA cycle. Glucose-derived and external pyruvate contributed more carbons to citrate, fumarate, and malate in MB1_UF cells, using both PDH and PC (Fig. 5E and F; Supplementary Fig. S5B). Conversely, more glutamine derived carbons were incorporated into TCA cycle metabolites in MB1_LF cells, in both the oxidative and reductive direction (Fig. 5E and G; Supplementary Fig. S5B). Accordingly, glutamine deprivation reduced mitochondrial oxygen consumption rate and total cellular ATP levels exclusively in these cells (Fig. 5H). However, the phenotype was flexible as switching from glutamine to pyruvate supply in MB1_LF cells not only reverted low pyruvate entry in the TCA cycle to levels found in MB1_UF cells (see Fig. 5E and F; Supplementary Fig. S5B) but also rescued respiration and ATP levels (see Fig. 5H).

**Figure 6.**
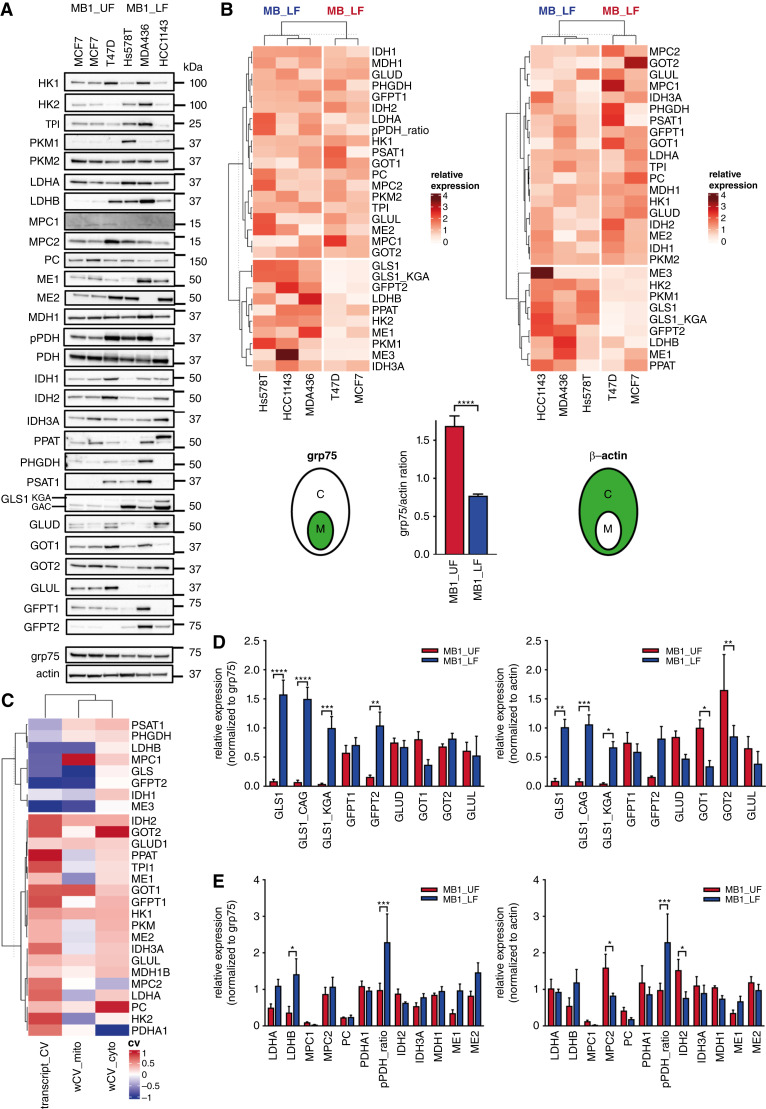
Protein expression profiles match transcriptomic switches. **A,** Western blot analysis of 29 metabolic enzymes across five cell lines representing MB1_UF and MB1_LF switch positions and cell states. Representative blots from *n* ≥ 3 independent experiments, used for quantification of signals, normalizing density values to either grp75 or β-actin. **B, D, **and** E,** Clustering and quantitative analysis of enzyme expression levels obtained from **A**. Values were separately normalized to either mitochondrial (left) or cytosolic (right) markers, grp75 or β-actin. The comparison of mitochondrial abundance based on the grp75/β-actin ratio is shown at the bottom in **B**. Heatmaps are based on a pairwise, robust to outliers distance measure of the mean protein expression values. The k-means clustering. **D** and **E,** Two-way ANOVA with uncorrected Fisher LSD multiple comparison tests. **C,** Heatmap and k-means clustering of transcript CV values of the 29 enzymes from the METABRIC dataset (see Figs. 1 and 2, positive values predicting higher expression of genes in MB1_UF and negative values corresponding to higher expression in MB1_LF) and scaled protein expression levels (wCV) averaged across all cell lines, normalized to grp75 (wCV_mito) or β-actin (wCV_cyto). ns, nonsignificant, *P* > 0.05; *, *P* ≤ 0.05; **, *P* ≤ 0.01; ***, *P* ≤ 0.001; ****, *P* ≤ 0.0001).

Finally, we assessed whether the metabolic transcriptome pattern and the functional metabolic phenotype is linked via protein levels of components of the affected metabolic pathways. Protein expression levels of 29 metabolic enzymes clustered according to switch positions (UF and LF) across the cell lines (Fig. 6A–C), corresponding to CV values. Overall, expression of most metabolic enzymes followed the higher mitochondrial abundance in the MB1_UF state (grp75/β-actin ratio). Those with the largest observed difference are involved in the malate–aspartate shuttle (GOT1, GOT2) and pyruvate utilization (MPC2, pPDH ratio, IDH2; Fig. 6D and E; Supplementary Fig. S6). In contrast, the set of enzymes expressed at higher levels in the LF supported its key characteristics, such as increased glutamine utilization (GLS1, GFPT2), together with aerobic glycolysis (HK2, PKM). Close correlation of metabolically key transcript and protein levels indicated that transcriptional regulation dominates pathway activities.

### Metabolic transcriptome patterns are independent predictors of pathological features and clinical outcome of breast cancer

**Figure 7.**
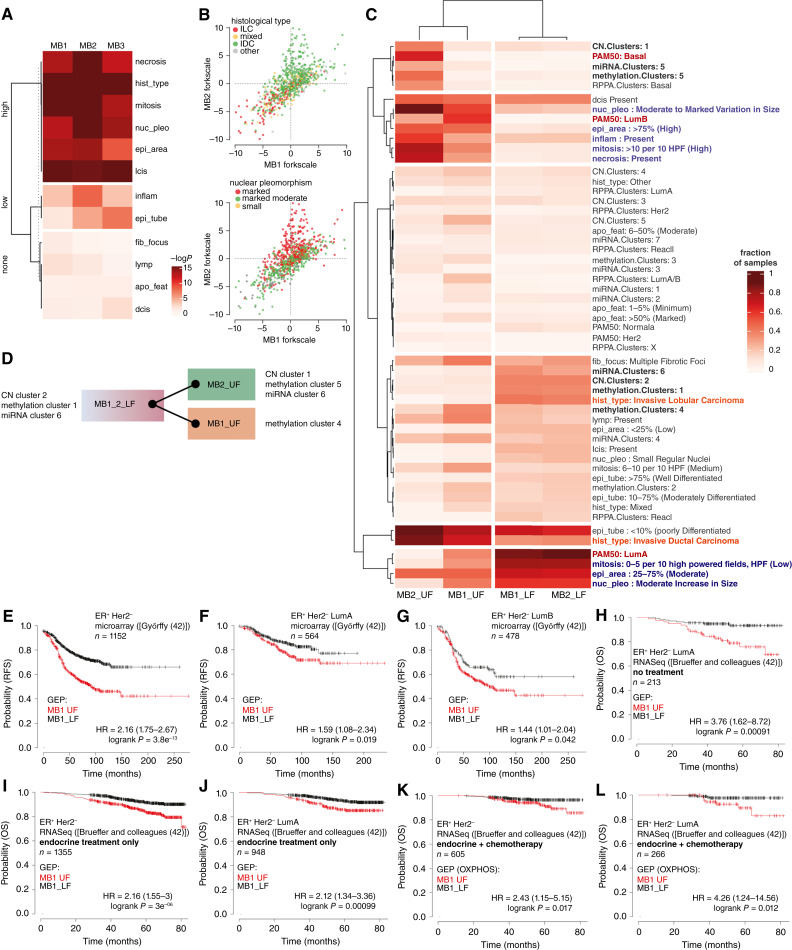
Pathological and clinical predictions from metabolic gene switches. **A,** The χ^2^ distribution of discrete histological features across the upper, lower, and non-fork samples of the three biclusters (MB1–MB3). Heatmap shows k-means clustering of χ^2^ test −log_10_*P* values, dividing the histological parameters into three groups: high, low, and no association with the bicluster. **B,** Visualization of histological feature distribution on the MB1 and MB2 switches. Classifiers of histological tumor type (top) and degree of nuclear pleomorphism are overlaid onto two-dimensional distribution plots of TCGA samples. The axes represent the range between the UF and LF of each bicluster. **C,** Clustering of histological and genetic features of samples belonging to the MB1 and MB2 switches. Fractions of samples for each feature were calculated and clustered with a custom robust to outliers distance measure function and k-means clustering. Genetic features representative of each switch position are highlighted (bold), Pam50 categories (red), and histology types and features (blue and orange, respectively). **D,** Key genetic and transcriptional architecture of the MB1 and MB2 switches. **E–L,** Survival analysis of samples in the MB1 switch. Kaplan–Meier survival plots and Cox proportional hazard analysis results from ref. 43 (**E–G**) and ref. 42 (**H**–**L**) are shown, generated by the kmplot.com tool (43). MB1_UF and MB1_LF bicluster (**E–J**) and OXPHOS (**K** and **L**) patterns (Supplementary Table S1) were used to stratify subsets of ER-positive, Her2-negative samples as indicated on the panels.

To assess the clinical relevance of the bicluster based classification, we tested the correlation of switch positions with genetic, biological, pathological, and clinical data in the TCGA and METABRIC datasets. Among the parameters in TCGA-BRCA (Supplementary Table S4; ref. 39), a group of features varied significantly between switch states (Fig. 7A). Assessing the fraction of samples having each histological and genetic feature, all UF switch positions were found associated with attributes of more aggressive tumors, such as frequent nuclear pleomorphism (Fig. 7B and C; Supplementary Fig. S7A) as opposed to the MB12_LF samples, which showed low proliferation and were enriched in tumors within the LumA intrinsic subtype and invasive lobular carcinoma histology. MB1_UF and MB2_UF samples showed increased proliferation, but they diverged by enrichment of diverse intrinsic subtypes and the underlying genetic and transcriptional constitution (CN variation, methylation, and miRNA clusters; Fig. 7D). Features in MB3_UF samples showed high overall similarity to MB12_LF but lacked enrichment in any specific genetic or epigenetic cluster (Supplementary Fig. S7B).

Finally, we asked whether metabolic switch positions in the MB1 bicluster can predict the clinical outcome of ER positive breast cancer. The MB1 switch includes almost exclusively ER positive tumors, splitting them in two groups, which partly overlap with the LumA, LumB, and normal-like subtypes (see Supplementary Fig. S2C). ER positive tumors overall are sensitive to antihormonal therapy, but adjuvant chemotherapy is usually recommended against tumors progressing to lymph nodes. However, only a fraction of these patients respond to this distressing treatment, motivating large recent efforts to develop gene expression pattern based methods to identify ER positive tumors responding to chemotherapy (40, 41). Thus, we assessed the effect of the MB1_UF and MB1_LF gene expression patterns on survival in the METABRIC and two independent breast cancer datasets (42, 43). The overall survival of patients with MB1_LF pattern expressing tumors was significantly better than the MB1_UF group (Supplementary Fig. S7C). The relative risk of relapse-free survival was similarly reduced in the ER positive, Her2 negative receptor subgroup independently of the lymph node status (Fig. 7E). Moreover, the MB1_LF gene expression pattern using the top correlating gene sets identified patients with significantly improved survival in both the LumA and LumB subgroups (Fig. 7F and G), indicating that the bicluster based classification improves the prediction value of the currently utilized Pam50-based system (44–46).

Data from the SCAN-B trial (42) allowed us to assess overall survival up to 7 years of the ER positive, Her2 negative subgroup following endocrine and chemotherapy treatment. Similar to the other datasets, the MB1_LF pattern indicated better survival even in nontreated or only endocrine-treated patients (Fig. 7H–J). Moreover, in patients receiving adjuvant chemotherapy, MB1_LF gene expression patterns of the OXPHOS and glutamine metabolism pathways distinguished a group with near 100% 5-year survival rate in contrast to 80% in the MB1_UF group (Fig. 7K; Supplementary Fig. S7D). These effects were independent of the tumors’ Pam50 status (Fig. 7J and L; Supplementary Fig. S7E). The data indicate that the MCbiclust classification can potentially predict treatment response in the most prevalent cases of breast cancer.

## Discussion

Pattern recognition, subgroup discovery and dimensionality reduction techniques often apply biclustering approaches to understand extensive and complex GEPs, underlying phenotype or cell state switches (47). MCbiclust is optimized for discovery of such large-scale, anticorrelating patterns (21, 23). Using the method, here we identified multistate GEP switches, defining novel breast cancer subtypes. The additional analysis pipeline assigned bioenergetic and metabolic phenotypes to the gene sets discovered. These metabolic and mitochondrial gene sets were strongly coregulated with a larger fraction of the transcriptome, revealing regulatory pathways underlying the metabolic adaptation during the differentiation of the cell of origin of tumor types. Robustly coregulated gene sets indicate common function, which likely is the basis of their power to predict tumor biology, histology, and ultimately clinical survival, the most compelling feature of the analysis. While the computed and experimental data properly validated the method for use on any large heterogeneous transcriptomic dataset, general predictions and specific results from the breast cancer dataset require thorough interpretation.

The relationship between transcriptomic patterns and the predicted features is undoubtedly not linear. Transcript levels of individual enzymes and metabolic fluxes are not always directly related (48), and apart from enzyme transcript and protein abundance, metabolic activity is fundamentally determined by substrate fluxes (49). However, computational models of metabolic networks have been shown to benefit from transcriptomic data integration, leading to the development of a series of tools capable of deducting verifiable metabolic properties (Supplementary Table S5). Here we did not seek to establish such a direct, linear transcript-to-flux link. Rather, we argued that long-term adaptive regulation of metabolism according to cell state is reflected in the coregulation (correlation) of large fractions of the transcriptome, including metabolism related genes. Examining the gene set patterns, as detailed in Materials and Methods, indicated alterations in metabolic fluxes. Ultimately, the predicted metabolic phenotypes were experimentally verified.

Breast cancer GEPs are being used to define intrinsic (PAM50) subtypes (44, 45) and integrated clusters (25, 50), but classification is further progressing by integrating histological data and emerging subtypes (27, 39, 51). Metabolic heterogeneity across known subtypes has also been explored [for recent reviews see (1, 52–54)]. However, studies are often limited to single pathways and comparison of broad tumor types without specific PAM50 definition (e.g., ER positive vs. triple negative). In contrast, MCbiclust uses a reverse and unbiased approach, covering the full spectrum of existing subgroups, based on bulk mitochondrial and metabolic transcriptome patterns. The approach thus enables discovery of previously unnoticed distinctions between subgroups and connections between metabolic pathways. For example, while glutamine utilization of triple negative tumors is well documented (55), MCbiclust indicated that high transcript levels of GLS, GFPT2, and PSAT1 are a common feature of all MB12_LF samples, comprising a range of PAM50 categories (Basal, LumA, and LumB). We show that glutamine in these cells is crucial for OXPHOS, associated with slower proliferation rates. In contrast, the highly proliferative MB1 and MB2_UF (comprising LumB, Basal, and Her2) groups show high PPAT levels, indicating glutamine usage for purine synthesis (36). Our results also give new insight into the role of OXPHOS in breast cancer. Currently, high OXPHOS activity is considered as a general feature of ER positive luminal tumors (53). However, we found the highest overall respiratory complex subunit transcript levels in MB1_UF and MB2_UF groups (subsets of Basal, Her2, LumB subtypes) in contrast to MB12_LF (subsets of LumA and Normal subtypes), and verified the finding experimentally. Thus, MCbiclust gave a bird’s eye view of bioenergetic activity across breast cancer, and clearly associated elevated OXPHOs levels with tumor aggressiveness.

Finally, our clinical survival analysis based on the MB1 bicluster provided compelling results. Although the analysis was performed in a retrospective cohort, the relative risk in the MB1_LF and MB1_UF groups is too large to have arisen by chance. The data additionally suggest that the survival prediction may be superior to that provided by intrinsic subtyping. ER positive tumors included in this analysis are sensitive to endocrine therapy as a group. The additional use of adjuvant chemotherapy has been widely recommended for patients with axillary lymph node involvement. However, only a fraction of patients benefit from this distressing treatment, motivating GEP-based methods to identify chemotherapy responsive groups (40, 41). The excellent outcome of patients in the MB1_LF despite other adverse prognostic features raises the possibility that patients in this group could safely avoid chemotherapy. In contrast, the poor outcome of the MB1_UF group demonstrates a need for additional or alternative approaches to treatment for these patients.

## Supplementary Material

Supplementary Figure Legends and Tables list Supplementary Figure Legends and Tables list

Figure S1 Supplementary Figure S1

Figure S2 Supplementary Figure S2

Figure S3 Supplementary Figure S3

Supplementary Figure S4 Supplementary Figure S4

Figure S5 Supplementary Figure S5

Supplementary Figure S6 Supplementary Figure S6

Figure S7 Supplementary Figure S7

Resource table Resource table

Supplementary Table S1 Supplementary Table S1

Supplementary Table S2 Supplementary Table S2

Supplementary Table S3 Supplementary Table S3

Supplementary Table S4 Supplementary Table S4

Supplementary Table S5 Supplementary Table S5
