# Supplementary material for: Multistate Gene Cluster Switches Determine the Adaptive Mitochondrial and Metabolic Landscape of Breast Cancer
Source: Cancer Res. 2024 Jun 26;84(17):2911–25. doi: 10.1158/0008-5472.CAN-23-3172 (PMC11372374; doi:10.1158/0008-5472.CAN-23-3172)
Supplement: Supplementary Figure Legends and Tables list [file can-23-3172_supplementary_figure_legends_and_tables_list_suppsd.pdf]

Supplementary Figure Legends and Tables list

Figure S1

A-C. Identification and initial characterisation of biclusters from the METABRIC dataset. Silhouette analysis of biclusters identified using the MB1 (**A**) and MB2/MB3 (**B**) genesets (see methods). **C.** Comparison of whole transcriptome CV values between all biclusters found. Density plots of the distribution of CVs across the transcriptome are shown for each bicluster in the central plots. Nuclear encoded mitochondrial genes are shown in red, non-mitochondrial genes are in cyan. The red dashed square indicates the three biclusters identified as independent, and carried over for further analysis.

D-E. Identification of the MB1-MB3 biclusters in independent datasets. **D** shows comparison of whole transcriptome CV values from METABRIC, TCGA-microarray, TCGA-RNAseq and Oslo2 datasets. Density plots of the distribution of CVs across the transcriptome are shown for each bicluster/dataset in the central plots. Nuclear encoded mitochondrial genes are shown in red, non-mitochondrial genes are in cyan. **E** shows the MB1 switch in 2D distribution by PC1 and ranking index in the four different dataset. Pam50 classifiers are overlaid to show the similarity of sample distribution in the different switches (biclusters). The result is representative of all three (MB1-MB3 biclusters).

F. Average gene group expression values in the upper and lower switch position in the MB1 switch in all datasets (METABRIC, Oslo2, TCGA-microarray, TCGA-RNAseq and TCGA-RRPA). Gene group1 (left panel) and gene group2 (right panel) are the two anticorrelated gene groups discovered by MCbiclust, as shown in Figure 1A. Accordingly, gene group1 shows positive correlation, and higher expression in upper fork samples as compared to the lower fork where conversely, group2 shows positive correlation and higher expression. The direction is arbitrarily set and harmonised between the biclusters. Turkey's box and whisker plots of the mean of the average gene group expression values across the indicated sample group (Lower, Upper Forks). The result is representative of all three (MB1-MB3) biclusters.

Figure S2

A. Mapping of highly scoring metabolic pathways from the METABRIC dataset on the global metabolic network. The MB1_UF (left panel) vs MB1_LF switch (right panel, blue) is represented. Pathways where any of the enzyme transcripts had a CV over 0.5 (MB1_UF, orange monocolor scale between CV = 0.5-1.0) or under -0.5 (MB1_LF, blue monocolor scale between CV = -0.5 - -1.0) are highlighted.

B. Visualisation of bicluster switches based on CV values of individual genes in the METABRIC breast cancer dataset analysis in the indicated metabolic pathways. In the left panels, genes are ranked by the MB1 CV values and directly compare the MB1 and MB2 biclusters. Right panels show the ranked MB3 values.

C. Overlap between classification of METABRIC breast cancer samples by switch positions (MB1-3 UF and LF) and PAM50 intrinsic subtypes.

Figure S3.

Statistical analysis of PARADIGM parameters across switch positions. Average PARADIGM values were calculated for each gene in each P cluster (Table S2), and compared between samples of each switch position. Kruskall-Wallis non-parametric test with Holm-corrected pairwise Dunn test was applied.

Figure S4.

A. Distribution of different cell-of-origin gene expression scores across the MB1 and MB2 switches.
B. Statistical analysis of cell-of-origin scores across switch positions. Kruskall-Wallis non-parametric test with Holm-corrected pairwise Dunn test was applied.

Figure S5.

A. Heatmap of fractional enrichment (FE) of ^{U13}C-glucose derived carbons in isotopologues of key metabolites analysed in MB1_LF (left panel) and MB1_UF (right panel) cells.
B. Overall carbon contribution into key metabolites following ^{U13}C-glucose (top left panel), ^{U13}C-glutamine (top right panel) and ^{U13}C-pyruvate (bottom panel) labelling in in MB1_LF and MB1_UF cells under different conditions (see Figure 5A).

Figure S6.

Quantitative analysis of enzyme expression levels obtained from western blot analysis (Fig. 6A**).** Values were normalised to either mitochondrial (left panel) or cytosolic (right panel) markers, grp75 or β-actin. 2-way ANOVA with uncorrected Fisher's LSD multiple comparison tests.

Figure S7.

A. Visualisation of histological feature distributions on the MB1 and MB2 switches (see Figure 7B). Classifiers of mitotic activity (left panel), fraction of epithelial area (middle panel) and presence of inflammation (right panel) are overlaid onto 2D distribution plots of TCGA samples along the axes representing the scale between the upper (UF) and lower (LF) forks of each bicluster.
B. Clustering of histological and genetic features of samples belonging to the MB3 switch. Fractions of samples for each feature were calculated and clustered with a custom robust to outliers distance measure function and K-means clustering.
C-E. Survival analysis of samples in the MB1 switch. Kaplan-Meier survival plots and COX proportional hazard analysis results from METABRIC (**C**) and (46) (**D, E**) are shown using the survival R package and kmplot.com tool (47), respectively. MB1_UF and MB1_LF bicluster (**C**) and glutamine metabolism (**D, E**) patterns (Table S1) were used to stratify subsets of ER positive, Her2 negative samples as indicated on the panels.

Supplementary Tables list S1-4

Table S1. **METABRIC data: sample characteristics and gene sets**

Table S**2. PARADIGM concepts and clusters**

Table S3**. Breast cancer cell line scoring results**

Table S4**. TCGA BRCA sample classification data with histological, genetic and transcriptomic features**

Table S5**. Methods for exploring transcriptome-metabolome relationships**
