# Supplementary figures and images for: Multistate Gene Cluster Switches Determine the Adaptive Mitochondrial and Metabolic Landscape of Breast Cancer
Source: Cancer Res. 2024 Jun 26;84(17):2911–25. doi: 10.1158/0008-5472.CAN-23-3172 (PMC11372374; doi:10.1158/0008-5472.CAN-23-3172)

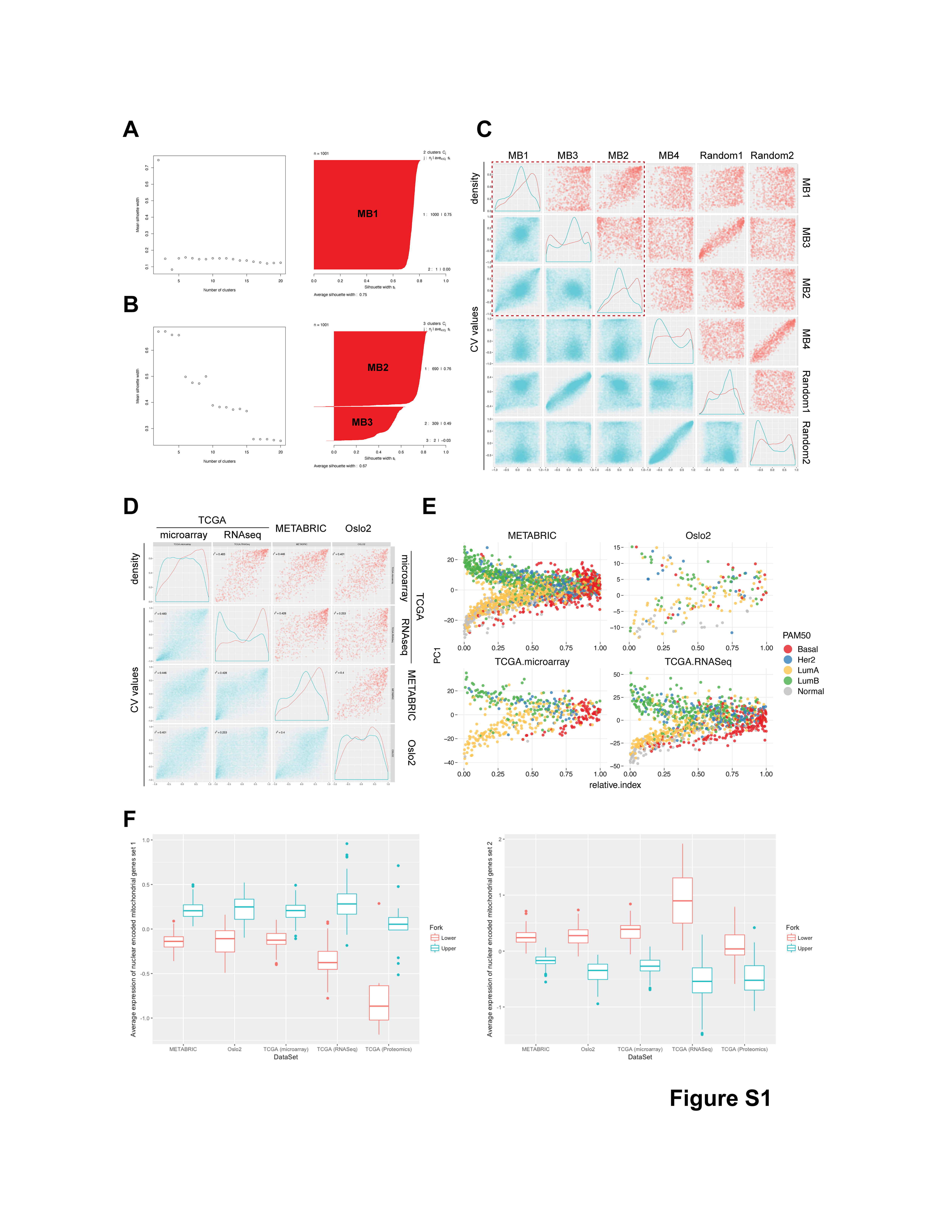

Supplement: Figure S1 — Supplementary Figure S1 [file can-23-3172_figure_s1_suppsf1.png]

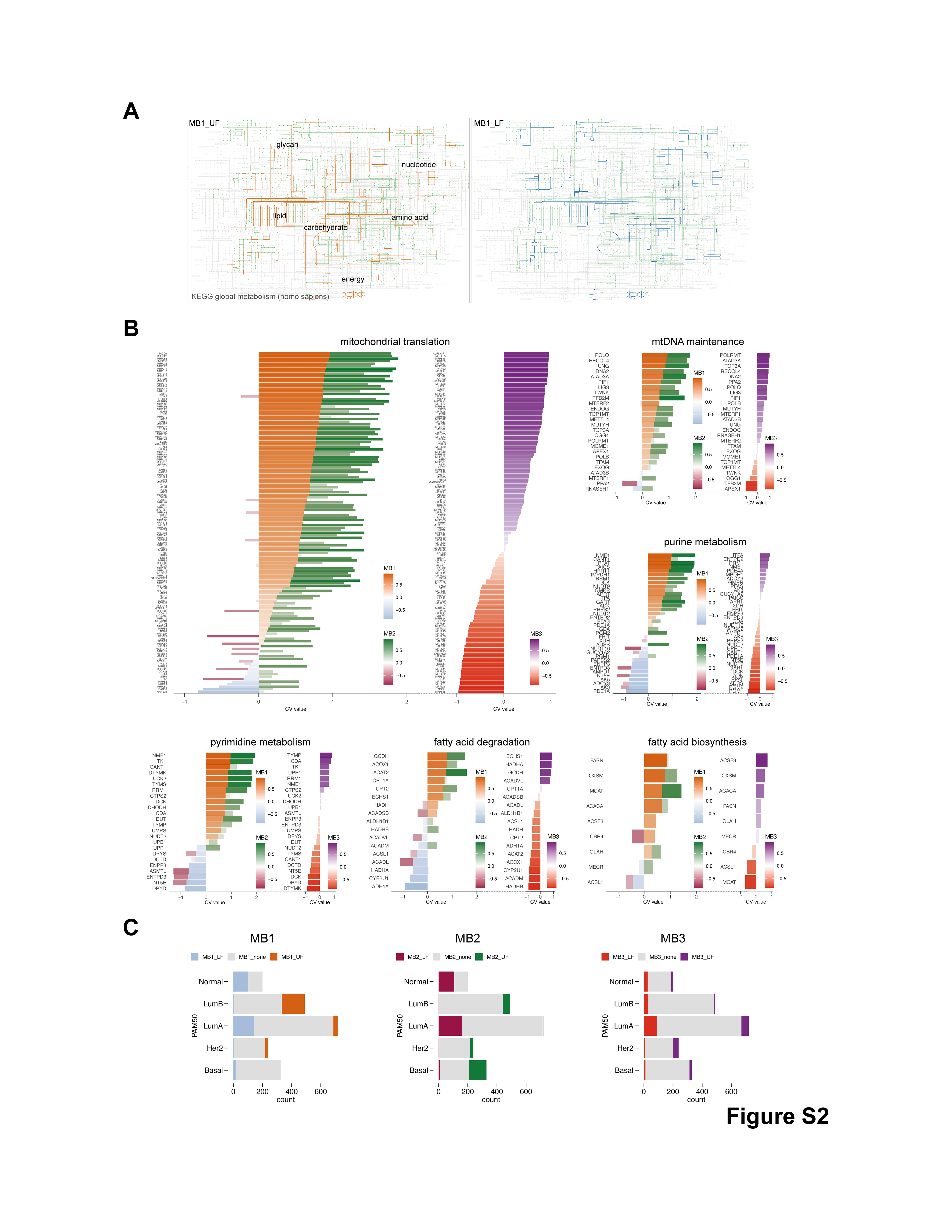

Supplement: Figure S2 — Supplementary Figure S2 [file can-23-3172_figure_s2_suppsf2.png]

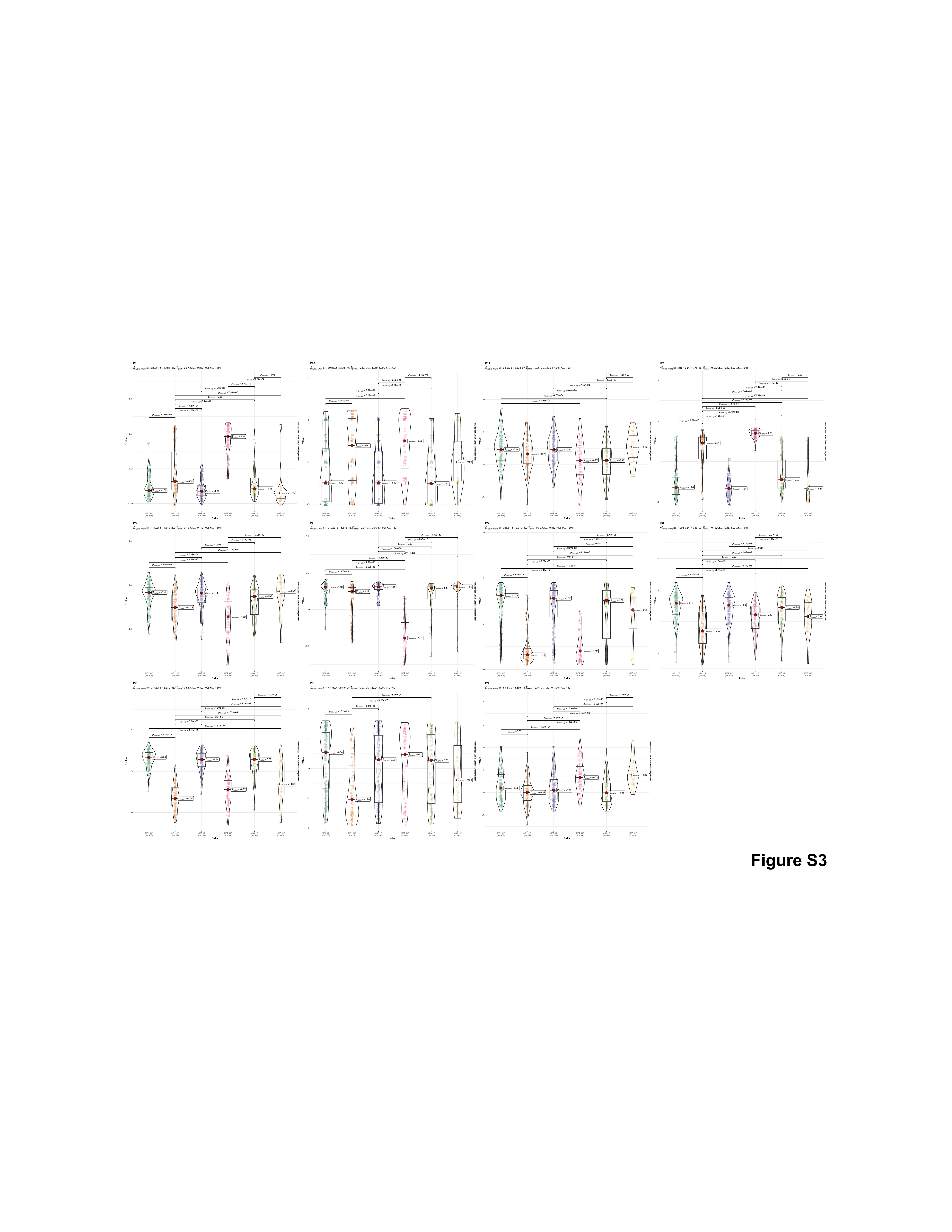

Supplement: Figure S3 — Supplementary Figure S3 [file can-23-3172_figure_s3_suppsf3.png]

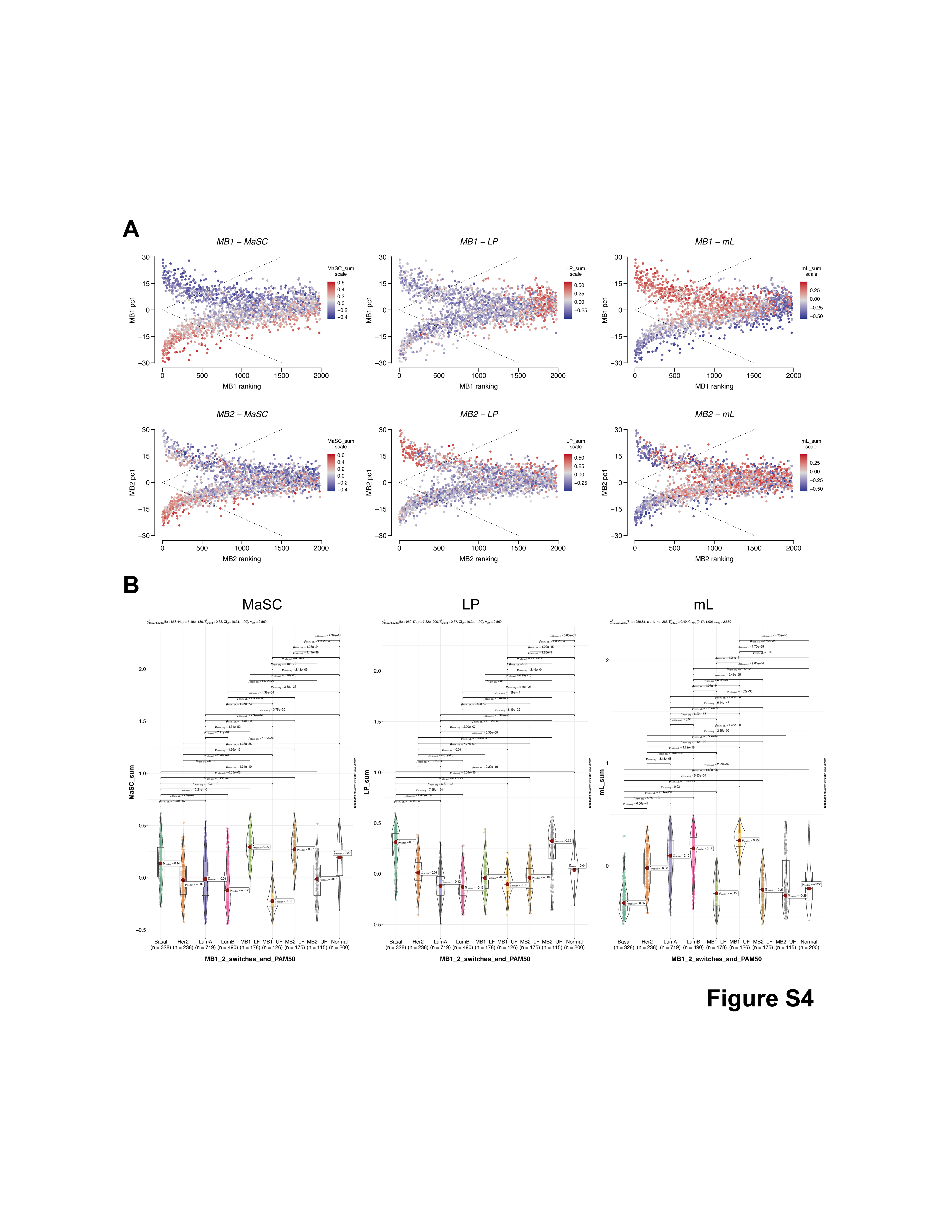

Supplement: Supplementary Figure S4 [file can-23-3172_supplementary_figure_s4_suppsf4.png]

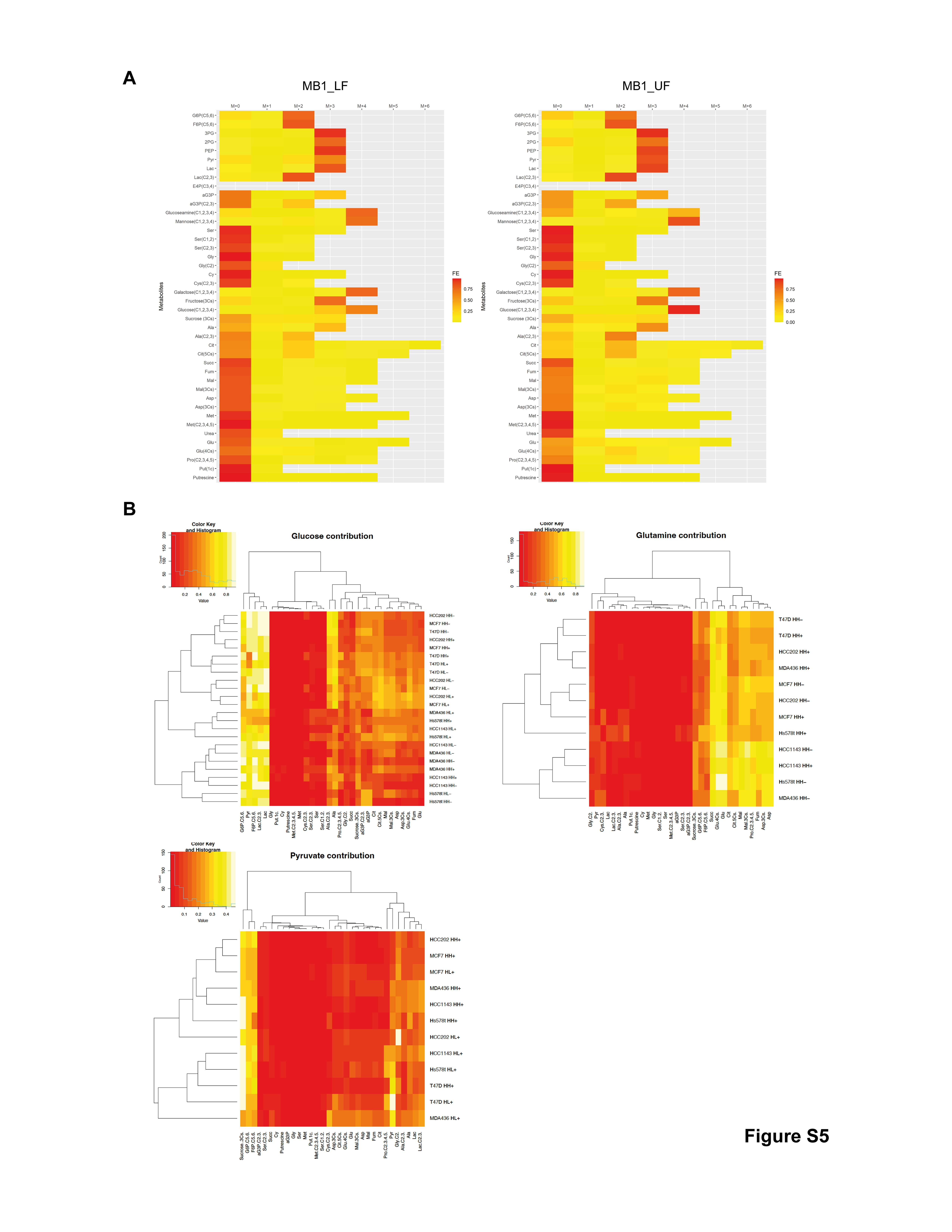

Supplement: Figure S5 — Supplementary Figure S5 [file can-23-3172_figure_s5_suppsf5.png]

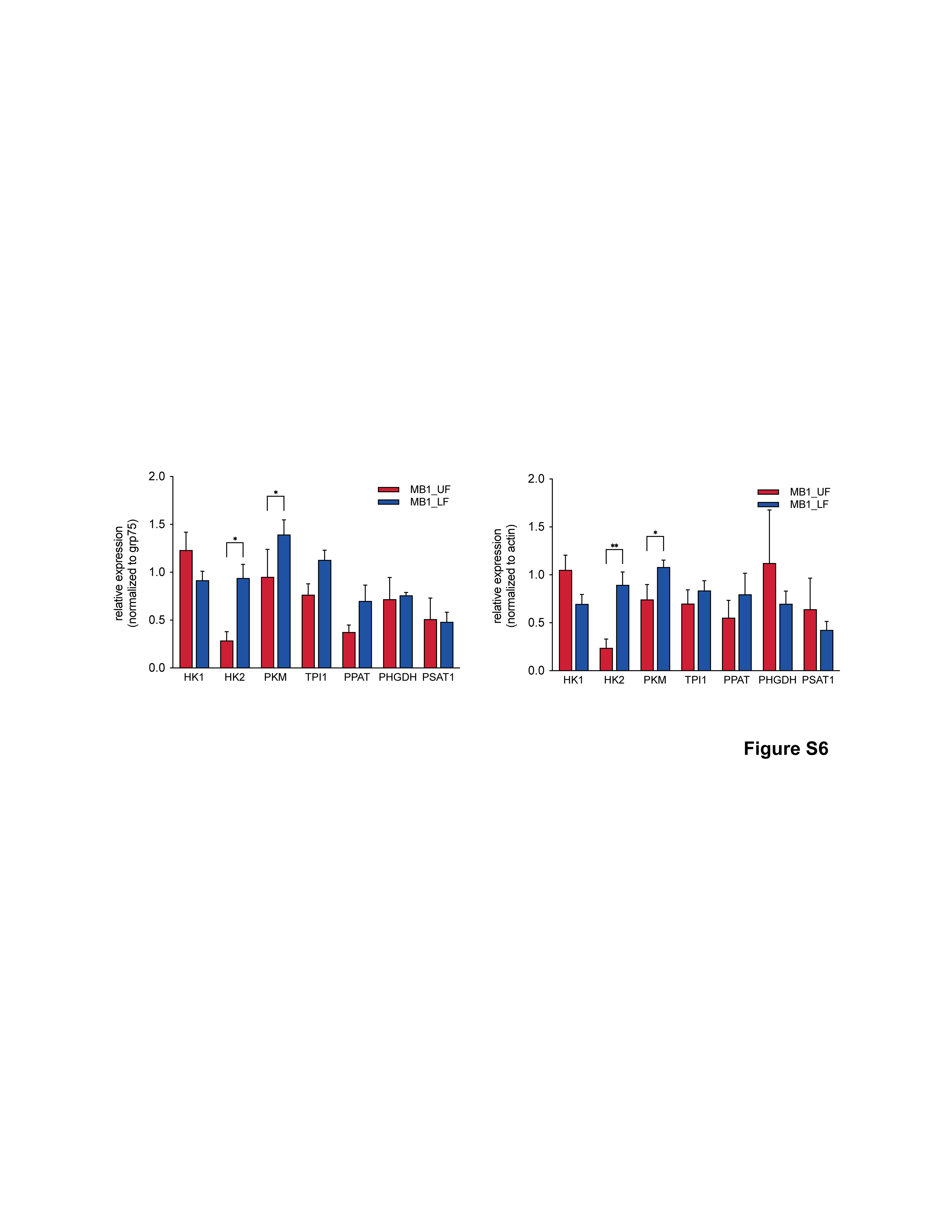

Supplement: Supplementary Figure S6 [file can-23-3172_supplementary_figure_s6_suppsf6.png]

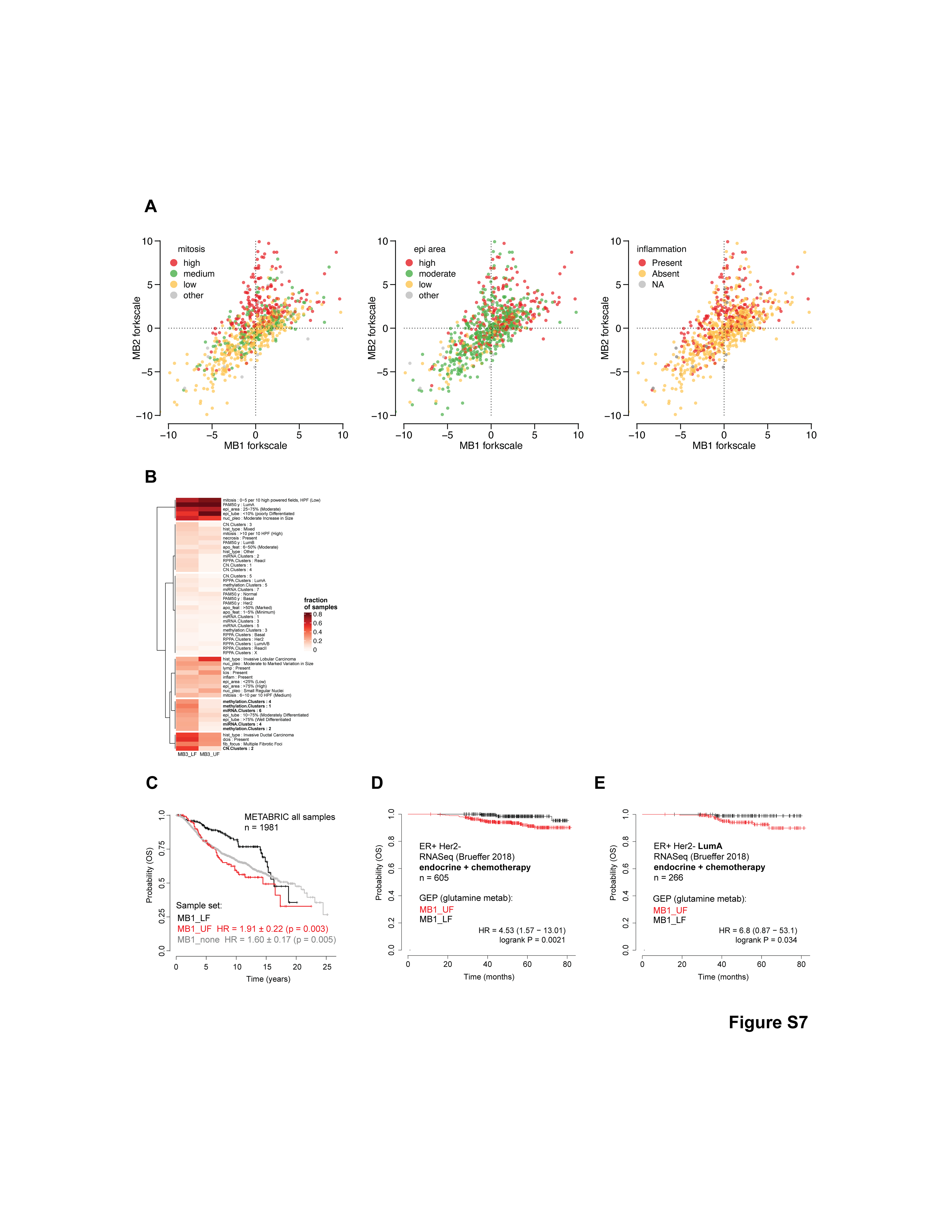

Supplement: Figure S7 — Supplementary Figure S7 [file can-23-3172_figure_s7_suppsf7.png]
