# Supplementary material for: Multistate Gene Cluster Switches Determine the Adaptive Mitochondrial and Metabolic Landscape of Breast Cancer
Source: Cancer Res. 2024 Jun 26;84(17):2911–25. doi: 10.1158/0008-5472.CAN-23-3172 (PMC11372374; doi:10.1158/0008-5472.CAN-23-3172)
Supplement: Resource table [file can-23-3172_resource_table_suppst.pdf]

Resources

REAGENT or RESOURCE	SOURCE	IDENTIFIER
Antibodies		
Rabbit monoclonal anti-Hexokinase 1	Cell Signaling Technology	Cat# 2024, RRID:AB_2116996
Rabbit monoclonal anti-Hexokinase 2	Cell Signaling Technology	Cat# 2867, RRID:AB_2232946
Rabbit polyclonal anti-Triosephosphate isomerase –N-terminal	Abcam	Cat# ab135532
Monoclonal antibody anti-Glyceraldehyde-3-phosphate dehydrogenase	Millipore	Cat# MAB374, RRID:AB_2107445
Rabbit monoclonal anti-Pyruvate kinase M1 isofrom	Cell Signaling Technology	Cat# 7067, RRID:AB_2715534
Rabbit monoclonal anti-Pyruvate kinase M2 isoform	Cell Signaling Technology	Cat# 4053, RRID:AB_1904096
Rabbit monoclonal anti-Lactate dehydrogenase A	Cell Signaling Technology	Cat# 3582, RRID:AB_2066887
Rabbit monoclonal anti-Lactate dehydrogenase B	Abcam	Cat# ab53292, RRID:AB_2234531
Rabbit polyclonal anti-BRP44L (MPC1)	Abcam	Cat# ab74871, RRID:AB_1523226
Rabbit monoclonal anti-MPC2	Cell Signaling Technology	Cat# 46141, RRID:AB_2799295
Mouse monoclonal anti-Pyruvate dehydrogenase E1-alpha subunit	Abcam	Cat# ab110330, RRID:AB_10858459
Rabbit polyclonal anti-pyruvate dehydrogenase E1-alpha subunit (phospho S293)	Abcam	Cat# ab92696, RRID:AB_10711672

Rabbit polyclonal anti-IDH1	Proteintech Group	Cat# 12332-1-AP, RRID:AB_2123159
Mouse monoclonal anti-IDH2	Abcam	Cat# ab55271, RRID:AB_943793
Rabbit polyclonal anti-IDH3A	Abcam	Cat# ab58641, RRID:AB_2295799
Rabbit polyclonal anti-glutaminase	Abcam	Cat# ab93434, RRID:AB_10561964
Rabbit monoclonal anti-GLUD1	Abcam	Cat# ab166618, RRID:AB_2815030
Rabbit polyclonal anti-GOT1	Proteintech Group	Cat# 14886-1-AP, RRID:AB_2113630
Rabbit polyclonal anti-GOT2	Proteintech Group	Cat# 14800-1-AP, RRID:AB_2247898
Rabbit polyclonal anti-ME1	Abcam	Cat# ab97445, RRID:AB_10679994
Rabbit monoclonal anti-ME2	Abcam	Cat# Ab139686
Rabbit monoclonal anti-PCB	Abcam	Cat# ab128952, RRID:AB_11144490
Rabbit polyclonal anti- 3PGDH (PHGDH)	Santa Cruz Biotechnology	Cat# sc292792
Mouse monoclonal anti-PPAT	Abcam	Cat# ab128116, RRID:AB_11143951
Rabbit polyclonal anti-PSAT1	Proteintech Group	Cat# 20180-1-AP, RRID:AB_10665948
Rabbit monoclonal anti-MDH1	Abcam	Cat# ab180152
Mouse anti - GLUL	BD Biosciences	Cat# 610518 RRID:AB_397880
Rabbit monoclonal anti-GFPT1 [EPR4854]	Abcam	Cat# ab125069, RRID:AB_10975709

Recombinant anti-GFPT2	Abcam	Cat# ab190966, RRID:AB_2868470
Total OXPHOS rodent antibody cocktail	Abcam	Cat# ab110413, RRID:AB_2629281
Mouse monoclonal anti-Actin	Santa Cruz Biotechnology	Cat# sc-56459, RRID:AB_830981
Rabbit polyclonal anti-beta tubulin	Santa Cruz Biotechnology	Cat# sc-9104, RRID:AB_2241191
Rabbit polyclonal anti-grp75	Santa Cruz Biotechnology	Cat# sc-13967, RRID:AB_647720
Chemicals, Peptides, and Recombinant Proteins		
[U-^{13}C] glucose	Cambridge Isotope Laboratories, Inc.	CLM-1396
[U-^{13}C] glutamine	Cambridge Isotope Laboratories, Inc.	CLM-1822
[U-^{13}C] pyruvate	Cambridge Isotope Laboratories, Inc.	CLM-1575
Pyruvate	Thermo Fisher Scientific	Cat# 11360070
Glutamine	Thermo Fisher Scientific	Cat# 25030081
Glucose	Thermo Fisher Scientific	Cat# A2494001
Normocin	InvivoGen	ant-nr-1
Hoechst 33342	Thermo Fisher Scientific	Cat# 62249
Tetramethylrhodamine, Methyl Ester, Perchlorate (TMRM)	Thermo Fisher Scientific	Cat# T668
Quant-iT™ PicoGreen™ dsDNA Assay Kits and dsDNA Reagents	Thermo Fisher Scientific	Cat# P11495
Rotenone	Sigma-Aldrich	Cat# R8875
Carbonyl cyanide 4-(trifluoromethoxy)phenylhydrazone (FCCP)	Sigma-Aldrich	Cat# C2920
Antimycin A from Streptomyces sp.	Sigma-Aldrich	Cat# A8674

Oligomycin A	Sigma-Aldrich	Cat# 75351
DMEM, high glucose, pyruvate	Thermo Fisher Scientific (Gibco)	Cat# 41966029
DMEM, high glucose, glutaMAX supplement, pyruvate	Thermo Fisher Scientific (Gibco)	Cat# 31966021
DMEM, no glucose, no glutamine, no phenol red	Thermo Fisher Scientific (Gibco)	Cat# A1443001
Critical Commercial Assays		
Glutamine/Glutamate-Glo Assay	Promega	Cat# J8022
CellTiter-Glo Luminescent Cell Viability Assay	Promega	Cat# G7570
Seahorse XF Cell Mito Stress Test Kit	Agilent	Cat# 103015-100
Experimental Models: Cell Lines		
MCF7	ATCC	HTB-22
T47D	ATCC	HTB-133
Hs578T	ATCC	HTB-126
MDA-MB436	ATCC	HTB-130
HCC1143	ATCC	CRL-2321
Source Data		
METABRIC	Synapse: syn1757063	https://doi.org/10.7303/syn1688369
TCGA	NCBI dbGaP	phs000178.v11.p8
OsloVal	Synapse: syn1710395	https://doi.org/10.7303/syn1688370
Software and Algorithms		
R v4.2.0	R Foundation for Statistical Computing	https://www.r-project.org/

MCbiclust 1.22.0	Bentham et al.(1)	https://doi.org/doi:10.18129/B9.bioc.MCbiclust
ComplexHeatmap	Gu(2)	https://doi.org/doi:10.18129/B9.bioc.ComplexHeatmap
GSVA 1.46.0	Hänzelmann et al(3)	https://doi.org/doi:10.18129/B9.bioc.GSVA
Fiji	Schindelin et al.(4)	https://imagej.net/software/fiji/
Prism 9.0	GraphPad Software	https://www.graphpad.com/
Other		
SnakeSkin Dialysis Tubing, 3.5K MWCO, 35 mm dry I.D., 35 feet	Thermo Fisher Scientific	Cat# 88244
Seahorse XF96 cell culture microplates	Agilent	Cat# 102416
Falcon® 96-well Black/Clear Flat Bottom TC-treated Imaging Microplate	Corning Life Sciences	Cat# 353219

1. Bentham RB, Bryson K, Szabadkai G. MCbiclust: a novel algorithm to discover large-scale functionally related gene sets from massive transcriptomics data collections. Nucleic Acids Res. 2017;45:8712–30.

2. Gu Z. Complex heatmap visualization. Imeta [Internet]. Wiley; 2022;1. Available from: https://onlinelibrary.wiley.com/doi/10.1002/imt2.43

3. Hänzelmann S, Castelo R, Guinney J. GSVA: gene set variation analysis for microarray and RNA-seq data. BMC Bioinformatics. 2013;14:7.

4. Schindelin J, Arganda-Carreras I, Frise E, Kaynig V, Longair M, Pietzsch T, et al. Fiji: an open-source platform for biological-image analysis. Nat Methods. 2012;9:676–82.
